# Genomic signatures and correlates of widespread population declines in salmon

**DOI:** 10.1038/s41467-019-10972-w

**Published:** 2019-07-05

**Authors:** S. J. Lehnert, T. Kess, P. Bentzen, M. P. Kent, S. Lien, J. Gilbey, M. Clément, N. W. Jeffery, R. S. Waples, I. R. Bradbury

**Affiliations:** 1 Fisheries and Oceans Canada, Northwest Atlantic Fisheries Centre, 80 E White Hills Rd, St. John’s, Newfoundland, A1C 5X1 Canada; 2 Biology Department, Dalhousie University, 6050 University Avenue, Halifax, NS B3H 4R2 Canada; 3 Centre for Integrative Genetics (CIGENE), Department of Animal and Aquacultural Sciences, Faculty of Biosciences, Norwegian University of Life Sciences, Ås, 1430 Norway; 4 Marine Scotland Science, Freshwater Fisheries Laboratory, Faskally, Pitlochry PH16 5LB UK; 5 Centre for Fisheries Ecosystems Research, Fisheries and Marine Institute of Memorial University of Newfoundland, 155 Ridge Rd, St. John’s, NL A1C 5R3 Canada; 6 Labrador Institute, Memorial University of Newfoundland, 219 Hamilton River Rd, Happy Valley-Goose Bay, NL A0P 1E0 Canada; 7 Fisheries and Oceans Canada, Bedford Institute of Oceanography, 1 Challenger Dr, Dartmouth, NS B2Y 4A2 Canada; 8 Northwest Fisheries Science Center, National Marine Fisheries Service, National Oceanic and Atmospheric Administration, Seattle, WA 98112 USA

**Keywords:** Evolutionary genetics, Population genetics, Biodiversity, Conservation genomics

## Abstract

Global losses of biodiversity are occurring at an unprecedented rate, but causes are often unidentified. Genomic data provide an opportunity to isolate drivers of change and even predict future vulnerabilities. Atlantic salmon (*Salmo salar*) populations have declined range-wide, but factors responsible are poorly understood. Here, we reconstruct changes in effective population size (*N*_e_) in recent decades for 172 range-wide populations using a linkage-based method. Across the North Atlantic, *N*_e_ has significantly declined in >60% of populations and declines are consistently temperature-associated. We identify significant polygenic associations with decline, involving genomic regions related to metabolic, developmental, and physiological processes. These regions exhibit changes in presumably adaptive diversity in declining populations consistent with contemporary shifts in body size and phenology. Genomic signatures of widespread population decline and associated risk scores allow direct and potentially predictive links between population fitness and genotype, highlighting the power of genomic resources to assess population vulnerability.

## Abstract (web summary)

The Atlantic salmon has suffered widespread population declines over the last century. Here, Lehnert et al. reconstruct changes in effective population size of 172 populations based on genomic linkage information revealing mostly temperature-associated population declines with over 60% of populations in decline since 1975.

## Introduction

Losses of biodiversity are rapidly occurring across the globe. Within the last century at least 200 vertebrate species have become extinct, and across taxa, numerous species are facing extinction risk^1,2^. Identifying causal factors of contemporary population decline is necessary for forecasting population changes and designing appropriate conservation actions. Genomic data provide a novel opportunity to investigate how populations have responded to change, identify mechanisms underlying these changes, and evaluate the adaptive potential and vulnerability of populations in the future^3,4^.

Increasingly, effective population size (*N*_e_), which is the evolutionary analogue of census size, is being considered as a relevant parameter in conservation genetics and management^5,6^. *N*_e_ represents the size of an ‘ideal’ population that would be expected to experience the same levels of inbreeding, genetic drift, and loss of genetic diversity as the population of interest^5,6^. *N*_e_ estimates can help assess the vulnerability of a population because decreases in *N*_e_ result in a greater influence of genetic drift relative to selection making small populations less capable of adapting genetically to environmental change^7,8^. Temporal genetic monitoring of *N*_e_ can provide powerful means to track populations changes over time^9^. However, with advances in analytical methods, temporal changes in contemporary *N*_e_ (~20–25 generations) can now be reconstructed from one sampling point using genomic data and genetic linkage information^10,11^, allowing characterisation of population trends without intensive long-term sampling. Quantifying changes in *N*_e_ can help identify factors which have shaped population abundance over time, with recent studies focused on environmental drivers of contemporary and future population changes^3,4^. However, the incorporation of high-density genomic data also provides an opportunity to circumvent identifying drivers of decline a priori and can instead characterise genomic regions key to population persistence.

Atlantic salmon (*Salmo salar*) is an ecologically, culturally and economically significant species that has experienced widespread population declines and extirpations throughout its North Atlantic range over the last century^12^. Current threats to Atlantic salmon populations include salmon aquaculture^13,14^ (e.g., farmed escapes), habitat alteration, pathogens, and climate change^15^. Though numerous factors can contribute to population losses, historical data necessary to quantify the role and interactions of multiple threats across broad scales are lacking^16^. Here, we use genomic data to reconstruct trends in population size across the range of Atlantic salmon and identify populations that have experienced significant declines in recent decades, allowing the investigation of environmental, anthropogenic, and genomic correlates of decline. We find population declines are temperature-associated across the range, and genomic regions associated with these declines relate to metabolic, developmental and physiological processes. The direct associations between genotype and population fitness found here can inform population vulnerabilities and enable conservation of adaptive diversity necessary for persistence of wild populations.

## Results

### Environmental and anthropogenic correlates of declines

**Fig. 1:**
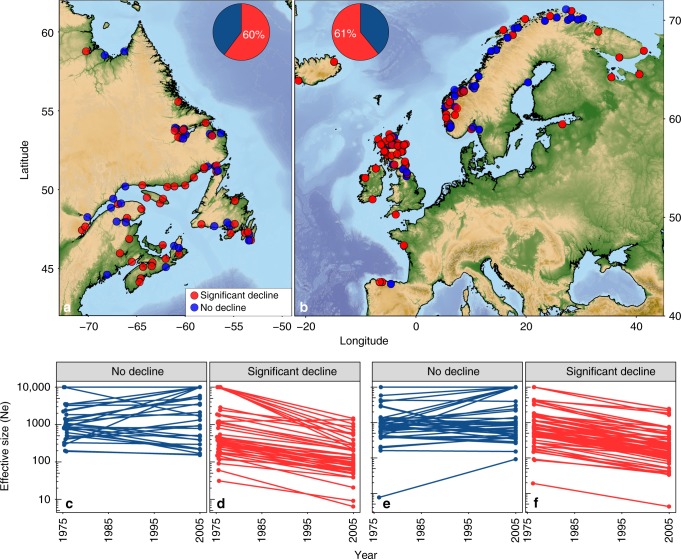
Changes in effective population size (*N*_e_) of Atlantic salmon across the range. Map of all salmon sampling locations in (**a**) North America (*n* = 73) and (**b**) Europe (*n* = 99) with significantly declining (red) and non-declining (blue) populations indicated by coloured points. Inset pie chart shows the proportion of populations assigned to each category, with panels (**c**–**f**) showing changes in *N*_e_ between 1975 and 2005 for non-declining (**c**, North America; **e**, Europe) and significantly declining (**d**, North America; **f**, Europe) populations. Any *N*_e_ estimates with large or negative (infinite) values were converted to 10,000 for this figure. Maps were generated using National Oceanic and Atmospheric Administration (NOAA) bathymetry data from the R package *marmap*^67^

Effective population size (*N*_e_) was reconstructed for 172 populations (*n* = 4493 individuals; Supplementary Data 1) across the Atlantic salmon range using a linkage-based method with the program LinkNe^10^, and validated using historical and contemporary samples for three locations (see Methods section). LinkNe^10^ bins loci based on linkage information where pairs of loci with similar recombination rates are binned together to estimate *N*_e_ at different generations in the past. For each bin of loci, the mean recombination rate (*c*) is used to estimate the number of generations (*t*) in the past (*t* = 1/2c). Assuming a generation time of 5 years for Atlantic salmon and a starting year of 2010 (see Methods section), estimates of *N*_e_ were calculated at four time points over approximately 25 generations. Focusing on recent declines (~6–7 generations), we found that 60% and 61% of populations in North America and Europe, respectively, showed significant declines in *N*_e_ since 1975 (Fig. 1).

**Fig. 2:**
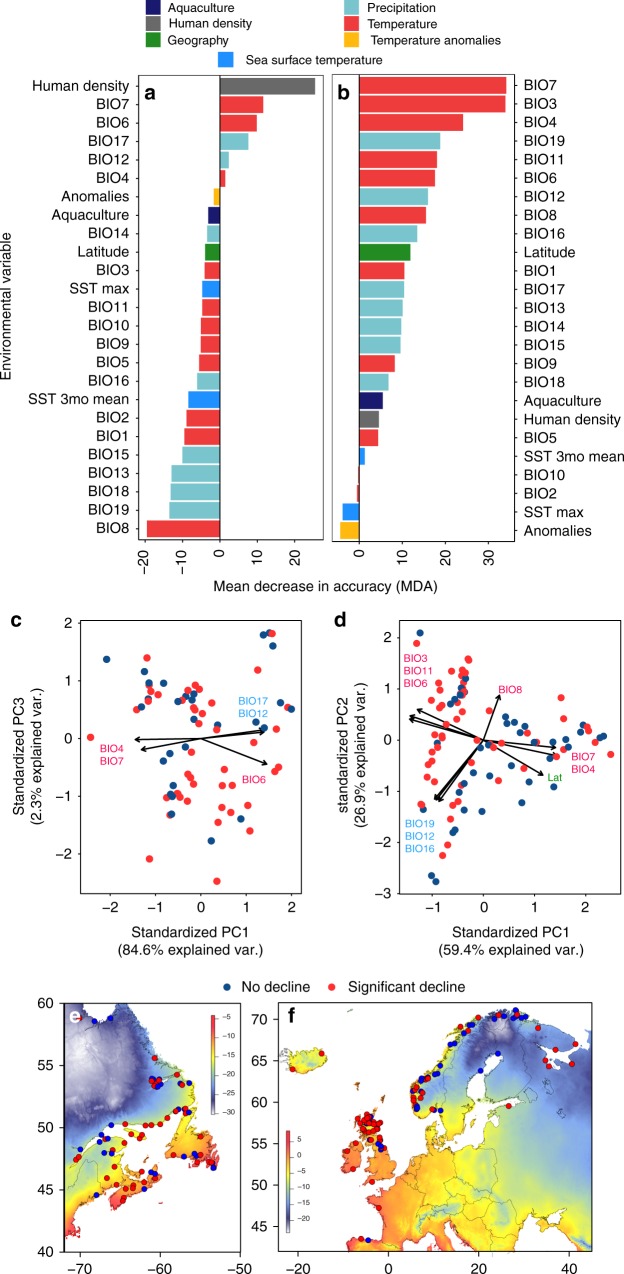
Environmental and anthropogenic associations with changes in effective population size of Atlantic salmon. Results of random forest (RF) classification for (**a**) North America and (**b**) Europe showing the environmental and anthropogenic variables arranged by decreasing mean decrease in accuracy (MDA). MDA values represent how the accuracy of RF decreases when the variable is excluded, thus higher values indicate greater importance. Codes for each variable are found in Supplementary Table 2. Principal component analyses (PCA) were performed to show relationships among top (positive) environmental variables within each continent (**c**, **d**) with panels showing PC axes correlated with population declines. Maps show variation in temperature (°C) measure (BIO6; minimum temperature in coldest month) that loaded high on significant PC axes, which, among other variables, correlated with declines in both continents (**e**, **f**)

Next, we investigated relationships between decline and environmental variables (climate in freshwater and coastal marine environments) and major anthropogenic threats^15^ (1-aquaculture intensity and 2-human density as a proxy for habitat disturbance^17^; see Methods section). Using random forest classification, top environmental and anthropogenic variables important for explaining variation in declines within each continent were identified (Fig. 2a, b). Top environmental variables were collapsed into principal components (enviro-PCs) (Fig. 2c, d) and used with top anthropogenic variables in generalised linear models. In North America, climate variables and habitat disturbance (human density) were important for explaining declines (Fig. 2a), where warmer winter temperatures (enviro-PC3, *p* = 0.04) significantly explained variation in population declines (GLM, *R*^2^ = 0.057). In Europe, population declines were explained by temperature, including greater variability (e.g., isothermality) and warmer winters (enviro-PC1, *p* = 0.016; Fig. 2e), as well as higher precipitation (enviro-PC2, *p* = 0.023) (GLM, *R*^2^ = 0.085). The influence of warmer temperatures at this broad-spatial scale is consistent with historical extirpations primarily occurring in southern regions, where other anthropogenic impacts are also stronger^12^ and these associations highlight the future threats of climate change. In other salmonids, findings of genetic constraints on upper thermal tolerance indicate that the adaptive potential of salmon might be limited under future climate scenarios^18^, suggesting that range shifts and additional local extirpations are likely in Atlantic salmon.

The importance of climate suggests that our results reflect broad-scale range-wide or continent-scale influences. Nonetheless, fine-scale factors influencing localised declines that are important at the individual river scale may be difficult to resolve. The observed geographic heterogeneity in decline may support a role for such small-scale local influences. For example, local factors could lead to changes within a single population that have no bearing on nearby populations, such as a new development project that alters suitable habitat^19^ or a natural (e.g., floods) or anthropogenic (e.g., toxic spills) catastrophic event that could lead to sudden changes within a river^20^. Furthermore, salmon populations are often considered to be locally adapted at fine spatial scales^21^ consistent with their strong natal philopatry and low straying rates^22^. Therefore, local adaptation can drive fine-scale differences between populations in important life history variation^21^ (e.g., proportion of multi-sea-winter adults or mature parr) that could influence the susceptibility of populations to losses over time^23^. Variation in the census size of populations may also influence our ability to detect significant changes^10^ and may further explain some fine-scale differences in decline patterns.

### Genomic correlates of population declines

**Fig. 3:**
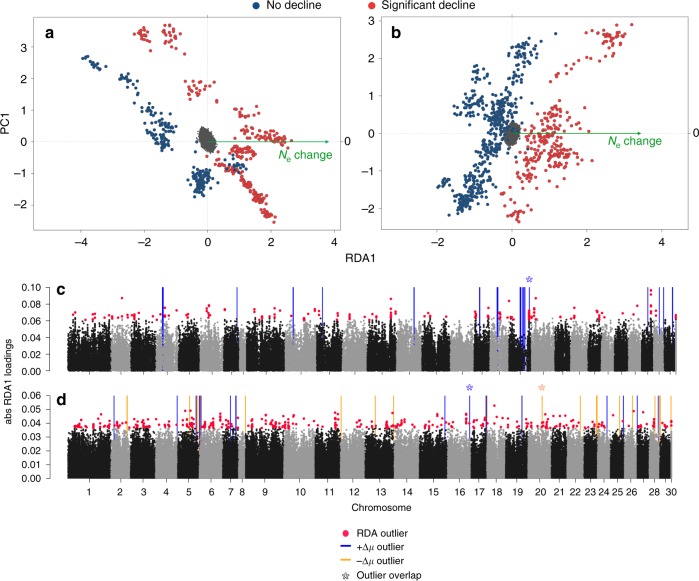
Genomic basis of population declines across Atlantic salmon using high-density genomic dataset. Redundancy analysis (RDA) separated significantly declining (red) and non-declining (blue) populations along the first axis in (**a**) North America and (**b**) Europe. Absolute RDA1 scores are presented in Manhattan plots for both continents (**c**, North America; **d**, Europe) with decline-associated loci (outliers) highlighted in red. Coloured lines represent regions showing significant changes in selective sweeps (∆µ) between declining and non-declining populations, where blue lines (+∆µ outlier) suggest an absence of selective sweeps in declining populations and orange lines (−∆µ outlier) suggest the presence of selective sweeps associated with declines. Asterisks (*) indicate regions with overlap between ∆µ and decline-associated SNPs (RDA) within each continent. SNPs on chromosome 30 represent those that did not align to known positions in the genome

Genome-wide data can also provide insight into genomic regions associated with population change. We found that higher-density genomic data (>99K and >181K SNPs in North America and Europe, respectively) from a subset of populations (*n* = 25 North American; *n* = 45 European) clearly separated significantly declining and non-declining populations (Fig. 3a, b). Redundancy analyses (RDAs) revealed significant polygenic associations with declines in *N*_e_ in North America (228 SNPs; Fig. 3c) and Europe (403 SNPs; Fig. 3d). Two SNPs were identified as decline-associated loci in both continents, and other decline-associated loci found in both continents were located in close proximity to each other. The SNPs associated with declines in both continents were found within the *trabd2a* (or *Tiki1*) gene on Ssa13. The gene plays a role in head formation^24^, and in Arctic charr (*Salvelinus alpinus*), a QTL in this region is associated with head shape^25^. In both continents decline-associated SNPs were also located near (<10 Kbp) three of the same genes, including *ST3GAL1-like* on Ssa03, *snd1* on Ssa17 and *GRID1-like* on Ssa01. *ST3GAL1-like* has been associated with mucus secretion (protection from pathogens) and migratory behaviour in salmonids^26^ and *snd1* is involved in various cellular functions including immune pathways^27^. *GRID1-like* belongs to a group of glutamate receptors which are involved in neurotransmission in the central nervous system and can play a role in learning and memory^28^. Functional enrichment of 15 and 11 gene ontology (GO) biological processes (*p* < 0.01; Supplementary Table 1) in North America and Europe, respectively, were found for annotated genes near decline-associated SNPs, including enrichment of metabolic and neural development processes, potentially highlighting morphological and physiological changes associated with declines range-wide.

Tests for selective sweeps revealed differences between declining and non-declining populations in patterns of presumably adaptive diversity at the continental scale in North America and across populations in Norway (Supplementary Fig. 3). A total of 28 and 17 genomic regions (1-Mbp windows) in North America and Europe, respectively, showed significant differences in the presence of selective sweeps, where evidence of selective sweeps was absent in declining populations (Fig. 3c, d). These differences in selective sweeps may suggest either a loss of adaptive diversity in declining populations or adaptation within non-declining populations necessary for persistence. A region displaying a loss of selective sweep on Ssa07 (48–49 Mbp) overlapped between continents (Fig. 3) and is homeologous to a decline-associated region (i.e., RDA) on Ssa17^29^. This genomic region has been linked to sexual maturation in Arctic charr^25^ and possible immune pathways^27^.

Within continents, several instances of overlap between genomic regions showing an absence of selective sweeps in declining populations and decline-associated loci using RDA were found. Within North America, one region on Ssa19 overlapped with 33 decline-associated SNPs (Fig. 3c) with the most significant SNP located next to flocculation protein (*FLO11-like*) that has been associated with ecotypes of Arctic charr^30^. In Europe, one region was associated with both differences in sweeps and population decline (Fig. 3d) and the highest loading SNP in this window was located near a gene with a role in development (*protocadherin-17-like* on Ssa16)^31^. Functional enrichment of GO biological processes associated with differences in selective sweeps were found for both continents (*p* < 0.01; Supplementary Data 2). The most significant processes in North America (*p* < 0.001) were related to cardiac development and regulation of cardiac muscle contractions, morphology, metabolism, and muscle adaptation. In Europe, highly over-represented biological processes (*p* < 0.001; Supplementary Data 2) were related to DNA replication and proofreading, sex organ development, metabolism and immunity. Only one biological process was significantly overrepresented in both continents and this was the cellular response to hydrogen peroxide which may play an important role in wound healing in teleosts^32^.

In Europe, 25 genomic regions showed an opposing pattern of selective sweeps, with signals of sweeps present only in declining populations (Fig. 3d). Sweeps in declining populations could indicate adaptive changes associated with disturbance. Highly enriched GO biological functions (*p* < 0.001; Supplementary Data 2) in these regions included those relating to metabolism, immunity, vision, neural and olfactory development, reproductive cycle, histone modification and regulation of heart rate. In addition, one region from this sweep analysis overlapped with decline-associated SNPs (RDA, Fig. 3d), where the highest loading SNP in the window was located in proximity to a gene related to development (*ADAMTS15* on Ssa20)^33,34^. The second highest loading SNP in the window was located near a thiamine receptor (*SLC19A3-l* on Ssa20). Thiamine deficiency has been suggested to play a role in widespread wildlife declines^35^ and has been implicated as a possible mechanism for historical losses of Atlantic salmon populations^36^.

All above functional associations highlight possible adaptive changes linked to morphology, physiology, or life history, consistent with long-term declines in body size and shifts in life history documented in wild Atlantic salmon populations^37,38^. These genomic signatures of decline are present across large latitudinal scales spanning heterogeneous environments, and thus the broad-scale nature of our analyses likely improves our ability to detect real decline-associated genomic variation rather than variation associated with geographic differences in selection. Although the majority of genomic changes were not parallel between continents, trans-Atlantic differences in underlying genomic architecture have been documented for many key traits related to domestication^39^, age-at-maturity^40^, and temperature-associated adaptation^41^.

### Predicting population vulnerability to declines

**Fig. 4:**
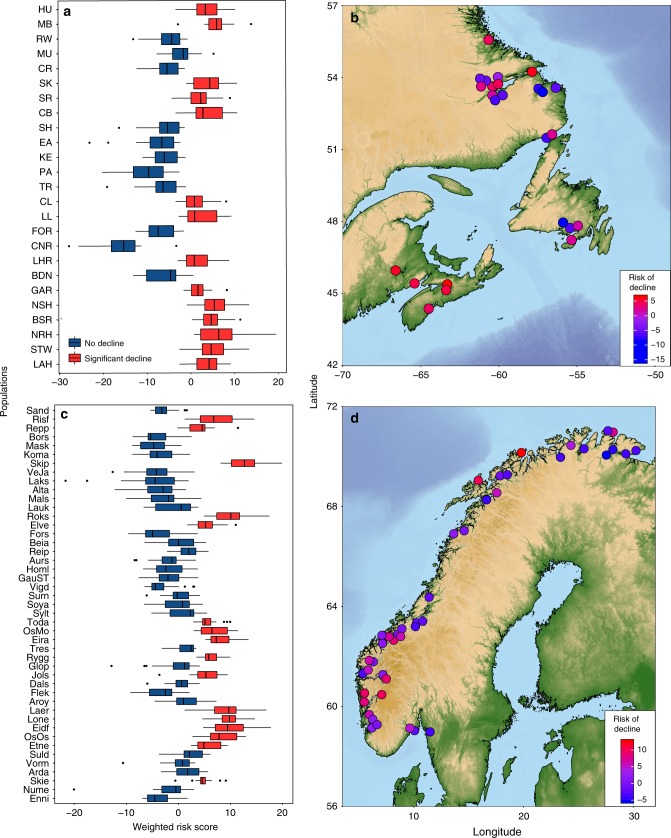
Predicting risk of declines across Atlantic salmon populations. Boxplots of weighted polygenic risk scores for population declines in (**a**) North America and (**c**) Europe calculated from decline-associated loci (RDA) in PredictABEL. Populations are arranged by latitude. For boxplots, centre line indicates median risk score, box limits represent upper and lower quartiles, whiskers indicate 1.5 × interquartile range, and points outside this range are outliers. Maps show mean score values for each population in (**b**) North America and (**d**) Europe. Maps were generated using National Oceanic and Atmospheric Administration (NOAA) bathymetry data from the R package *marmap*^67^

The clear link between population fitness (i.e., decline) and genotype identified here was used to calculate weighted polygenic risk scores^42^ (i.e., risk of decline). Using a small panel of genome-wide decline-associated loci based on RDA, our results highlight populations at greatest risk of decline, including non-declining populations that might soon be at risk, such as Mulligan River (MU) in Canada and Suldalslågen River (Suld) in Norway (Fig. 4). In addition, our data suggest high repeatability for scores across >94% of populations when data from the population of interest are excluded in the construction of the risk model (see Methods section). Therefore, these methods may offer a novel tool for fisheries managers to identify populations most susceptible to future losses.

## Discussion

It has been over two decades since scientists first asked why there are not more Atlantic salmon in the wild^12^. Despite conservation efforts, our study supports existing evidence that populations continue to decline throughout the range. We found that environmental and anthropogenic factors correlated moderately with declines, and these factors likely represent drivers that influence declines in freshwater and coastal marine environments. At a broad range-wide scale examined in our study, temperature variables were more important than anthropogenic factors for explaining variation in population declines, although habitat alteration was also important for explaining variation in declines within North America. Across the range, declines were consistently associated with warmer winter temperatures which can influence early development in Atlantic salmon and may have implications for later life stages^43^. At finer geographic scales, specific local influences (e.g., aquaculture, dams, floods) likely play a role in local population change as well, though the effects are unlikely to be consistent at the continent or range scale examined here. In addition, unidentified factors in the ocean contribute substantially to mortality^16^ and likely reflect the unexplained variance in our model as data on marine migration and habitat use are currently limited.

Multifarious selective pressures have likely driven declines and our study shows, for the first time, genomic changes associated with declines across a species’ range. Decline-associated loci consistent between continents were located near genes that have been previously linked to head morphology, immunity, and migratory behaviour in salmonids^25–27^. Furthermore, a process related to wound healing in fishes^32^ was significantly overrepresented by genomic regions associated with changes in adaptive diversity in declining populations in both Europe and North America. Additional changes in adaptive diversity documented here in genomic regions linked to developmental, physiological and reproductive processes are consistent with the loss of large salmon and life history changes across many rivers^37,38,44^. The changes related to declines in *N*_e_ in our study highlight the vulnerability of >60% of salmon populations across the range. As *N*_e_ decreases, the effects of genetic drift can outweigh those of selection, and thus these declining populations may be more susceptible to loss in the future^7^. Overall, our work highlights the value and need for genomic resources in conservation management where these data can be utilised to understand the mechanisms driving population change and predict future vulnerabilities.

## Methods

### Salmon genotyping

Genotypes of salmon from North American and European populations were compiled from a previous study^41^ (available at: https://doi.org/10.5061/dryad.cv20d) from datasets that used either a 220,000 or 6000 SNP array with overlapping markers between arrays^45–48^ (see Supplementary Data 1). We also incorporated genotype data from Norwegian populations that used the 220,000 SNP array^49^ into our dataset resulting in a total of 99 European populations (*n* = 2858 individuals) and 73 North American populations (*n* = 1635 individuals) for our analyses. For our main *N*_e_ analysis, we excluded collection sites that were sampled prior to the year 2000. For 172 populations across the range, a total of 1278 SNPs were used for a linkage-based estimate of effective population size (*N*_e_) over time^10^. SNPs were determined based on overlap between datasets, minor allele frequency (MAF) >0.05 across the range, and whether linkage map information was available for both continents^50,51^.

To our knowledge, the various studies from which our genomic data are compiled did not aim to avoid rivers that could be impacted by salmon farming. Many sites include regions where the impacts of aquaculture have been investigated^13,52–54^. Therefore, prior to *N*_e_ analyses, populations located near aquaculture operations were analysed for evidence of farm introgression. Details of introgression analyses are provided in the Supplementary Note 1 and any individuals with evidence of aquaculture ancestry were removed from *N*_e_ analyses (see Supplementary Table 3). We did not attempt to identify natural migrants (strays) among sites within each population; however, we do not expect natural straying events to influence temporal trends in *N*_e_ examined here (see Supplementary Note 1). Genotype files were also screened for duplicates using gsi_sim^55^ and any redundant samples (i.e., no genotype mismatches) were removed (*n* = 7).

### Reconstruction of effective population size (*N*_e_) over time

LinkNe^10^ was used to reconstruct *N*_e_ for each population over time using default parameters of the program (bin size of 0.05 Morgans, allele cutoff frequency of 0.05, sample-size bias correction) and a time bin by generation. Loci were binned based on linkage using the average of the male and female linkage maps within each continent. Linkage maps of sexes were averaged because sex-specific differences in recombination rate occur in Atlantic salmon, where recombination in females is 1.38 to 2.22 times greater than in males^50,51^. The mean recombination rate (*c*) for each bin was used to estimate the number of generations (*t*) in the past (*t* = 1/2c) for each estimate of *N*_e_. The number of loci pairs used for estimates in each bin are provided in Supplementary Fig. 1. Although more pairs of loci are unlinked and thus contribute to calculations of recent *N*_e_ (Supplementary Fig. 1), LinkNe implements a sample-size bias correction^56^ that effectively corrects for these differences across bins^10^. We then assumed a generation time of 5 years^57–59^ and a starting year of 2010 to determine estimates of *N*_e_ at different time periods in the past. Assumptions regarding generation time and starting year do not influence estimates of *N*_e_, but do influence the time periods reported herein. Despite variation in sampling year (range of sampling year: 2000–2014; see Supplementary Data 1) and life history across populations (i.e., generation time), our estimates represent recent changes in contemporary *N*_e_ which may represent different time frames in different populations, but nonetheless represent the same number of generations in every population.

### Classification of declining versus non-declining populations

To assign populations as significantly declining or not declining, we used estimates of *N*_e_ with empirical 95% confidence intervals (CI) for values between the years 1975 (~6–7 generations ago) and 2005 (~1 generation ago). Populations were classified as significantly declining if *N*_e_ decreased and CIs did not overlap between 1975 and 2005. In cases where lower CIs were negative (i.e., indicating very large or infinite), we considered a population to be significantly declining if CIs included infinite values in 1975 but CIs were non-infinite in 2005 (i.e., unbounded to bounded *N*_e_ estimates). Populations that were not declining could include populations with a non-significant increase or decrease in *N*_e_ (i.e., no significant change) and populations with a significant increase in *N*_e_. We did not attempt to estimate the magnitude of decline because the true magnitude of decline cannot be confidently estimated, as historical estimates of *N*_e_ will be influenced by the effects of genetic drift in the recent samples that exhibit declines (see Hollenbeck^10^).

We acknowledge that differences in sample size among populations (see Supplementary Data 1) may influence precision of *N*_e_ estimates. Smaller sample sizes will reduce precision and lead to wider confidence intervals (infinite) and this is especially true when true *N*_e_ is large. Therefore, we expect that significant declines in *N*_e_ may be more difficult to detect in a large population with a smaller sample size^10^ and thus the number of populations that have significantly declined may be underestimated here. Nonetheless, we find no evidence to suggest that different sample sizes led to bias in our temporal trends in *N*_e_ (see Supplementary Note 2). In addition, differences in sampling strategy (i.e., age class sampled; see Supplementary Data 1) may also lead to bias in *N*_e_. However, we do not expect this to influence trends in *N*_e_ over time, unless age structure has changed substantially and differentially among populations. Although we cannot rule out this bias in our dataset, we find no evidence that age structure influenced our temporal analyses of *N*_e_ (see Supplementary Note 2).

To validate the above classification method, we compared *N*_e_ estimates using NeEstimator v2^60^ for three populations that had both historical (1997 or earlier) and recent (2007 or later) samples. Here, we examine whether temporal changes in *N*_e_ found using LinkNe with recent samples are consistent with temporal changes when *N*_e_ is estimated for two time points using historic and recent samples. *N*_e_ was estimated using the linkage-disequilibrium method for the same panel of 1238 SNPs with MAF cut-off of 0.05. *N*_e_ estimates and confidence intervals determined using the Jones et al.^61^ jackknife method implemented in NeEstimator were compared between historical and recent samples using the same criteria as above to classify populations as significantly declining or non-declining. Classifications using NeEstimator agreed with our classifications using LinkNe for all populations (see Supplementary Fig. 2), thus supporting our classification method.

### Environmental and anthropogenic drivers of declines

Environmental and anthropogenic variables were collected from publicly available sources (see Supplementary Table 2). A total of 19 bioclimatic variables of temperature and precipitation data (mean from 1970 to 2000) were accessed from WorldClim^62^ (resolution 2.5 arc-min). Sea surface temperature (SST; mean from 2002 to 2010) for months when salmon smolts would be moving into the marine environment (May–July) were extracted and averaged from MARSPEC^63^ and long term average of maximum SST (from 2000–2014) was extracted from Bio-ORACLE^64^. Mean annual global temperature anomalies averaged between the years 1975–2005 (resolution 0.5 degrees) was accessed from NASA Earth Observations (NEO) (https://neo.sci.gsfc.nasa.gov). For temperature anomalies, anomaly values for each year represent the difference in temperature (colder or warmer) for the same region relative to the average temperature between 1951 and 1980.

For all above measures, data were extracted for each site using latitude and longitude for each location. Site coordinates were shifted slightly if necessary. For example, for ocean variables, site coordinates were adjusted to near the river mouth (i.e., into the marine environment).

Human population density in 2000 (resolution 0.1 degrees) was also accessed from NASA NEO. Human population density was a proxy for habitat disturbance^17^ as we expect higher human densities to have greater anthropogenic impacts on habitat through increased human occurrence. Values were averaged from a larger grid (0.5**°** latitude × 0.5**°** longitude) around the site to capture human activity in the region surrounding the population. Data were log transformed for random forest analyses. To test the assumption that human density is an appropriate proxy for habitat disturbance, we used available data from North American populations that have been evaluated for impacts associated with habitat alterations^65^. Regional groups (designatable conservation units) were classified by a cumulative impact score of low (<5%), medium (5–30%) or high (>30%) based on the proportion of salmon affected by habitat alterations, such as municipal waste water, industrial effluents, dams, urbanisation, transportation infrastructure and other alterations (see ref. ^65^). For all regions with known cumulative impact classifications (*n* = 14 regions), we calculated the mean human density for salmon populations located within these regions (*n* = 26 populations total). We found that human density was significantly different among low, medium and high impact groups (Supplementary Fig. 4; Kruskal–Wallis, *X*^2^ = 7.49, df = 2, *p* = 0.024), where pairwise post-hoc comparisons revealed that the low impact group had significantly lower human density relative to the high impact group (*p*-adjusted = 0.04).

Atlantic salmon aquaculture site and year information were acquired from government resources, and an aquaculture intensity index (also previously referred to as propagule pressure^52^) was estimated for each population within continents using the AQpress function in R^52^. Previous work has demonstrated that this index is significantly associated with the number of farmed escapees found in rivers, as well as the proportion of aquaculture introgression in wild populations^52^. Data for North America were previously compiled^52^ between 2005 and 2015, with data on the presence and absence of each aquaculture site for each year. In Europe, we assessed data for only two countries, which represent the greatest producers of Atlantic salmon aquaculture in the region: Norway and Scotland^66^. In Scotland, we used all aquaculture sites that were registered since 2006 and/or were active in the last 3 years (source: http://aquaculture.scotland.gov.uk/data/site_details.aspx). In Norway, we used all sites that were registered since 2006 as no information was available prior to this year (source: https://kart.fiskeridir.no/akva). We acknowledge that more historical aquaculture data would be beneficial for these analyses provided the time frame examined for population decline. Nonetheless, we assume that the same geographic regions that are amenable to aquaculture presently would be amenable to aquaculture in the past. For example, in Canada, the same geographic regions in NB, NS and NL would be expected to harbour aquaculture sites in the past and present, although the intensity may change over time. In North America (*n* = 202 aquaculture sites), aquaculture intensity index was averaged across years as presence and absence of each aquaculture operation was known in each year. In Europe, we did not have data for each year for all aquaculture sites (*n* = 1080 aquaculture sites), therefore aquaculture intensity index was calculated assuming all sites were active at the same time (one year). Using the AQpress function^52^, latitude and longitude for sites were shifted to move sites slightly offshore to allow least cost distance calculations. In Europe, the AQpress function^52^ was modified to incorporate European bathymetry using the *marmap*^67^ package. Data were log transformed for random forest analyses. Although this metric of aquaculture intensity has been correlated with the number of escaping salmon and introgression in some regions^52^, here, we use the aquaculture intensity index to represent a relative measure of aquaculture activity near a river site. River sites that are in close proximity to many aquaculture operations will have higher aquaculture intensity index indicating a greater potential for interactions with aquaculture, including interactions with escaping salmon, aquaculture pathogens, pollution, and other effects on the local environment.

All 24 environmental and anthropogenic variables and latitude were included in random forest^68^ classification analysis to identify variables important for explaining population declines. All predictor variables were standardised and run using the R package *randomForest*^69^. The number of parameters sampled for each node split (mtry) was selected using the tuneRF function^69^ with 10,000 trees and stratified sampling with equal sample sizes for both groups for each run. All other parameters were set to default. The relative importance of each predictor was determined using the mean decrease in accuracy (MDA) averaged across runs. MDA values represent how the accuracy of RF decreases when the variable is excluded, thus higher MDA values indicate greater importance in the model and negative MDA values indicate that the incorporation of the variable reduces model accuracy. Therefore, within each continent, the top 10 predictors that were positive (contributed to model) were used for a generalised linear model (GLM). To account for correlations among environmental variables, environmental data were collapsed into principal components (PCs) using prcomp function in R. Anthropogenic factors (*n* = 2) were not collapsed into PCs. For our analyses, the first 3 environmental PCs (enviro-PCs) explained 99 and 95% of the environmental variation in North America and Europe, respectively and were thus used in subsequent models. We tested all three PCs and important anthropogenic variables (if identified by RF) in a GLM with binary response (decline or no decline) within each continent. For any significant enviro-PCs in the model, the importance of environmental variables contributing to declines was determined by loadings on PC axes.

Provided the different spatial and temporal scales of both environmental and anthropogenic data, we acknowledge the limitation of this data. Specifically, many of the variables are averaged data over multiple years, which could reduce our ability to identify a critical event in one year that contributed to population changes^70^ or identify factors that contributed to declines at different temporal scales^71^. High resolution data collected during the same time frame as the declines would be ideal; however, limited resources at fine scales (e.g., weather stations) restrict our ability to compile comparable and high resolution datasets at large spatial scales.

### Genomic basis of population decline

Redundancy analyses (RDAs) were used to investigate the genomic basis of population declines using high-density genomic markers. A subset of populations genotyped for a 220,000 SNP array was used for the analysis, which included 25 and 45 populations in North America^72^ and Europe^49^, respectively. We note that in some cases different individuals were genotyped for the same population between the 6K and 220K arrays (Supplementary Data 1). First, SNPs were filtered for quality where only SNPs that were considered high resolution in North American datasets were retained (185,883 SNPs). The European dataset was further filtered to remove any additional low quality SNPs specific to the European dataset (185,041 SNPs). Next, SNPs were filtered for minor allele frequency (MAF) of 0.05 within North America (99,224 SNPs), and the same subset of SNPs was used for Europe and subsequently filtered for MAF (181,425 SNPs). RDAs were performed using the R package *vegan*^73^ with change in *N*_e_ (significant decline vs. no decline) as a constraining factor and conditioned on geography (latitude) to explain variation in individual genotypes. Missing individual genotypes were replaced by the highest frequency genotype for the locus. Decline-associated SNPs were identified as SNPs with scores ±3 standard deviations (i.e., outliers) from the mean on the RDA axis^74^.

Within each continent, RAiSD^75^ was used to detect selective sweeps within declining and non-declining populations separately. To incorporate a larger number of markers for the analyses, we filtered the 220,000 SNP dataset for high quality SNPs within each continent and MAF of 0.01 within each continent (*n* = 149,263 SNPs in North America; *n* = 217,182 SNPs in Europe). RAiSD calculates the µ statistic (representing signatures of sweeps) across the genome from overlapping windows. To compare differences in selective sweeps between significantly declining and non-declining groups, we calculated the change in µ (∆µ) by subtracting µ in declining populations from µ in the non-declining populations, representing the difference in adaptive diversity between these groups. We calculated ∆µ in Europe and North America separately. Higher (positive) values of ∆µ indicate sweeps present in the non-declining populations and absent in the declining populations, which may indicate a loss of adaptive diversity in declining populations or a change in adaptive diversity in the non-declining populations underlying potential adaptation enabling population persistence. Lower (negative) ∆µ values indicate sweeps present in the declining populations and absent in the non-declining populations, which may indicate adaptive diversity that has been selected in declining populations in response to population disturbance. ∆µ was calculated for 1 Mbp windows and ∆µ values ± 3 standard deviations (SD) from the mean were considered significant outlier regions. These outliers were compared against outliers from the RDA by examining decline-associated SNPs that fell within 1 Mbp of the start or end of the ∆µ window. We allowed a 1 Mbp range outside of the ∆µ window given that windows calculated in RAiSD are based on a SNP-driven, sliding-window algorithm and thus the exact window size/position is not defined by the user and may be slightly offset^75^.

### Functional enrichment of biological processes

For both decline-associated SNPs (RDA) and sweep outlier windows, we examined functional enrichment of genes in these regions. We conducted gene ontology (GO) enrichment analysis using GO annotations in the Atlantic salmon genome from SalmoBase^76^. We first identified reference sets of genes for each analysis using BEDTOOLS^77^ where for the RDAs we extracted genes within 10 Kb of all SNPs used in the analysis and for the sweep analysis we extracted genes within all windows tested. Genes associated with outlier SNPs and within window regions were extracted in the same way. The R package topGO^78^ was then used to test for over-representation of GO biological processes using a node size of 5 and the ‘weight01’ algorithm to account for structural relationships among GO terms. We used an alpha level of 0.01 to determine significance.

### Calculation of polygenic risk scores for predicting risk of decline

PredictABEL^42^ was used to calculate weighted polygenic risk scores based on genotypes of RDA outliers using the riskScore function. The top 100 loci were selected based on their RDA loadings and physical distance, where the highest loaded SNP outliers in 1 Mbp windows were retained for the analysis. In total, 100 decline-associated loci were used for European analysis, but only 90 loci were used for the North American analysis after filtering based on physical distance. Model coefficients for each locus were determined by the fitLogRegModel function and weighted polygenic risk scores were calculated for all individuals to estimate risk of decline.

To test the repeatability of risk scores, scores were computed for individuals with the population of interest excluded from the construction of the risk model (i.e., leave-one-out approach). For each population, weighted risk scores with and without the population included in risk model were compared using a Mann–Whitney test. Alpha level was adjusted for comparisons in each continent based on the number of populations (i.e., 25 North America, 45 Europe). Across the range, only 6% of populations (*n* = 4) showed significant differences in risk scores when the population was excluded or included in the construction of the risk model (Supplementary Figs. 5 and 6). While no populations in Europe showed significant differences (all *p*-values > 0.04; Supplementary Fig. 6), four populations in North America had scores that were significantly different when the population was excluded or included in model construction (Supplementary Fig. 5). These populations were located in a region of southern Newfoundland and in Labrador, suggesting greater geographic coverage in these regions may help improve score repeatability in future studies.

### Reporting summary

Further information on research design is available in the Nature Research Reporting Summary linked to this article.

## Supplementary information

Supplementary Information

Supplementary Data 1

Supplementary Data 2

Reporting Summary

Description of Additional Supplementary Files

## Data Availability

All genotype data were compiled from previous studies. Genotype data for LinkNe analyses were accessed from 10.5061/dryad.cv20d^41^ and additional Norwegian genotype data^49^ were added 10.5061/dryad.23h4q. Additional genotype data from 220K SNP array were compiled from other studies^45,72^ (see 10.5061/dryad.4m5d5m9; 10.5061/dryad.93h33/1). Environmental and anthropogenic data files and genomic information for outliers (RDA and sweeps) are available at: https://github.com/SarahLehnert/SalmonDecline. No custom scripts were used in these analyses.
