# Supplementary material for: Genomic signatures and correlates of widespread population declines in salmon
Source: Nat Commun. 2019 Jul 5;10:2996. doi: 10.1038/s41467-019-10972-w (PMC6611788; doi:10.1038/s41467-019-10972-w)
Supplement: Supplementary file 1 — Supplementary Information [file 41467_2019_10972_MOESM1_ESM.docx]

**Supplementary Information**

**Genomic signatures and correlates of widespread population declines in salmon**

Lehnert et al.

**This PDF file includes:**

**Supplementary Notes**

Supplementary Note 1: Potential aquaculture introgression and straying

Supplementary Note 2: Sample size and age structure

**Supplementary Figures 1 to 11**

**Supplementary Table 1 to 3**

**Supplementary References**

**Supplementary Notes**

**Supplementary Note 1: *Potential aquaculture introgression and straying***

*Screening of individuals for aquaculture introgression*

Many of our sites include geographic regions where the impacts of aquaculture have been previously examined^1-5^. Therefore, prior to N_e_ analyses, populations located near aquaculture operations were analyzed for evidence of farm introgression. Introgression from aquaculture is possible in regions where salmon aquaculture occurs including Norway and Scotland in Europe, as well as in the Bay of Fundy region (New Brunswick [NB], Nova Scotia [NS]) and in southern Newfoundland (NL) in North America. Potential farm escapees or wild-farm hybrids were identified and removed from the dataset, and the methods for each region are detailed below. All Norwegian samples (n=45 populations) were previously screened for aquaculture introgression and any fish with possible aquaculture escapee ancestry were previously removed^6^. Samples from Scotland and North America were not previously screened for aquaculture introgression and therefore all samples from regions of intense aquaculture (see Supplementary Table 3) were screened for aquaculture introgression using Newhybrids^7^ as per a previous Atlantic salmon study^1^. Analyses were performed at the population level and follow a similar protocol across sites with small modifications within specific regions detailed below.

First, to explore assignment accuracy, simulations were run for each population in triplicate^8,9^ using the R package *hybriddetective*^9^ where local wild and farmed genotypes were used to simulate pure and multigenerational hybrid individuals (pure wild, pure farmed, F_1_, F_2_, backcross-wild, backcross-farm). For each population, a panel of 250 loci was chosen for subsequent analyses (detailed below). Simulated datasets were run in Newhybrids and we used *hybriddetective*^9^ to determine the accuracy of assignment. Accuracy represented the proportion of individuals that were correctly assigned to their respective genotype group (pure wild, pure farmed, F_1_, F_2_, backcross-wild, backcross-farm). To estimate the probability of an individual being a wild-farmed hybrid, we summed the posterior probabilities for assignments of all hybrid groups (F_1_, F_2_, BC-wild, BC-farm). Overall accuracy in hybrid identification using our simulations was high using a posterior probability threshold of >0.75 (Supplementary Table 3). In these simulations, pure types were assigned with an average accuracy of 97.5% across all populations (range: 86.1-100%) and hybrids were assigned with an average accuracy of 93.2% across populations (range: 82.8-100%). This approach was subsequently used to screen the various regions for farmed and hybrid individuals.

In Scotland, the majority of the sites in our study are located in the east whereas the majority of aquaculture operations are located in the west^10^. We expect that farmed introgression may be more likely at the seven sites that are located in western Scotland (latitude range: 56-59°) near intensive aquaculture operations (see Supplementary Figure 7). For Newhybrid analyses, we used a population of Norwegian farmed broodstock (genotype data from Dryad and used in our study^11^; <https://doi.org/10.5061/dryad.cv20d>) as a representative farmed population (BIS, n = 20) because the Scottish aquaculture strain originates from Norway^10^. BIS samples were previously obtained from the Ocean Science Center, Memorial University of Newfoundland and genotyped in previous study^11^.

Within each Scottish river population, a panel of 250 diagnostic loci were chosen using getTopLoc function in R package *hybriddetective*^9^ from a total of 1774 loci^11^. Using the genotype data for the top 250 loci, pure groups (wild and farm) and multigenerational hybrids (F1, F2, BC-wild, BC-farm) were simulated from the pure wild and pure farmed individuals (as above) using the freqbasesim_AlleleSample function in *hybriddetective*^9^. Simulated and real genotype data were run in Newhybrids using the R package *parallelnewhybrid*^12^. All Newhybrids runs were performed with 100,000 burnin and 500,000 sweeps.

The same methods were applied to the southern Newfoundland (NL) populations where aquaculture is present (5 sites) (Supplementary Figure 8). For NL analyses, we used farmed salmon originating from the Saint John River (NB) aquaculture strain that were previously sampled for use in other wild-farm hybrid studies in NL^8^. Two populations both originating from the same Saint John River strain were used^11^ (<https://doi.org/10.5061/dryad.cv20d>). A total of 1774 loci were used to generate a panel of 250 discriminatory loci for each population. Similar methods were applied to populations (5 sites) in the Bay of Fundy (BOF) region (NS and NB; Supplementary Figure 8) using data collected with either the 6K (GAK, STW) or 220K (NSH, NRH, BSR) SNP arrays depending on the river. To reduce the 220K datasets, loci within each dataset (one wild and farmed population) were filtered for MAF >0.05, high *F*_ST_ (>0.2), and more than 80% of individuals genotyped. A subsample of 2500 of these loci (highest *F*_ST_) was then used to generate the panel of 250 loci using getTopLoc function.

Individuals from all wild populations were primarily assigned to the pure wild group with some individuals being assigned to backcross-wild group (see Supplementary Table 3). For the LinkNe analyses, we removed all individuals from our dataset that had a 75% chance or greater of being a true hybrid or escapee.

*Screening of North American individuals for recent European introgression from aquaculture*

In addition to introgression from local aquaculture strains in North America, European salmon have also been previously farmed in the Bay of Fundy (BOF) and evidence of European introgression from aquaculture escapes has been found in the region^13^. Salmon genotyped using the 220,000 SNP array in our study (26/73 populations in North America) were investigated for European introgression in a previous study^58^. Although several populations in North America show evidence of historical introgression from secondary contact with European salmon (estimated to have occurred ~10,000 years ago), only a single individual out of >500 individuals sampled showed evidence of recent European introgression (second generation backcross)^14^. This individual was from the BSR (Big Salmon River, NB) population in the BOF region^14^ and was removed from the LinkNe analysis. Therefore, we expect the frequency of European introgression from aquaculture to be low in the BOF region in agreement with other work^15^ and provided that we have used a MAF >0.05 within each population for our N_e_ analyses (see Methods), we do not expect such low levels of European introgression (if present at all) to bias the trends in N_e_. To test this prediction, we performed analyses in LinkNe with the BSR population^14^ and we found that the inclusion or exclusion of the individual with European introgression did not change the classification of the population as significantly declining and thus did not influence the temporal trend in N_e_.

*Potential for natural straying events among rivers*

We expect that natural straying events are possible among nearby rivers; however, we have not attempted to identify or remove potential natural strays from our dataset, as they are unlikely to be easily detected among genetically similar populations and likely occur at low levels. Waples and England^16^ showed that under migration-drift equilibrium, the LD method estimates local N_e_ unless migration is high in genetic terms (5-10% or higher). Non-equilibrium (pulse) migration at high rates could produce a large bias. However, even if migration does lead to some bias, it should not affect trends unless the pattern of migration changes substantially. We do not expect patterns of natural migration (straying) events to change over time.

**Supplementary Note 2: *Sample size and age structure***

Data for our study were compiled from various studies that employed different sampling strategies and thus our populations vary in the life stage and sample size. The average number of individuals sampled per site was 26 individuals (Supplementary Data 1), where all sites had a minimum of 15 individuals sampled with the exception of a single site that had only 10 individuals (range: 10-40 individuals). The numbers of individuals sampled here are comparable to the empirical dataset used to evaluate the LinkNe program^17^, where a significant population decline was detected with only 14 individuals.

In North American samples, we found no significant difference between sample size of populations classified as significantly declining or not declining (Mann Whitney test: *U*= 544.5, *p*=0.278; see Supplementary Figure 9). In addition, population classification (non-declining vs. significantly declining) was not dependent on the life stage sampled (*X*^2^=3.14e-31; df=1, *p*=0.99; see Supplementary Figure 9). Nonetheless, we further investigated how age structure in adult samples could influence classification of decline. We utilized recently collected data from two populations with small (1-sea winter) and large (multi-sea winter) reproductively mature salmon. The respective age categories were analyzed separately and together in LinkNe following the same methods as all other analyses (see Methods). For both populations, the results show that the same classification was obtained regardless of which samples were incorporated in the analyses (Supplementary Figure 10). In addition, classifications were consistent despite 2-fold difference in sample size when cohorts were separated (n=24-26 versus n=50-52) (Supplementary Figure 10).

In Europe, sample size and life stage sampled varied by geographic region, where sites sampled in the UK generally had larger sample sizes and were collected as parr whereas sites in Norway generally had lower sample sizes and were collected as adults (see Supplementary Data 1 and Supplementary Figure 11). This disparity confounded our ability to directly investigate the role of sample size and life stage. Nonetheless, we separated samples based on life stage (parr [or unknown sampling] and adults), and within each life stage, there was no significant difference in sample size between significantly declining and non-declining populations (parr: Mann-Whitney *U*=267.5, *p*=0.19; adults: Mann-Whitney *U*=240.5, *p*=0.85; see Supplementary Figure 11). We included the parr with the unknown samples as these samples are likely juveniles, and no significant difference in sample size was found whether the unknowns were included or excluded.

# Supplementary tables

**Supplementary Table 1.** Gene ontology enrichment for genes located near (<10Kbp) outlier SNPs associated with changes in N_e_ based on redundancy analysis (RDA) in North America and Europe. The numbers of genes with GO annotations are provided for both the reference dataset (N_anno_) and outlier dataset (N_sig_) along with the expected number (N_exp_) based on a random distribution and significance (p-val) using Fisher’s exact test. An alpha level of 0.01 was used to determine significance.

| **GO ID** | **Description** | **N_anno_** | **N_sig_** | **N_exp_** | **p-val** |
| --- | --- | --- | --- | --- | --- |
| *RDA North America* | |  |  |  |  |
| GO:0010172 | embryonic body morphogenesis | 18 | 3 | 0.14 | 0.00034 |
| GO:0016337 | single organismal cell-cell adhesion | 844 | 13 | 6.56 | 0.00148 |
| GO:0050794 | regulation of cellular process | 7211 | 62 | 56.01 | 0.00162 |
| GO:0006538 | glutamate catabolic process | 8 | 2 | 0.06 | 0.00162 |
| GO:0042775 | mitochondrial ATP synthesis coupled electron transport | 21 | 3 | 0.16 | 0.00365 |
| GO:0010960 | magnesium ion homeostasis | 12 | 2 | 0.09 | 0.00374 |
| GO:0010842 | retina layer formation | 42 | 3 | 0.33 | 0.00416 |
| GO:0071108 | protein K48-linked deubiquitination | 15 | 2 | 0.12 | 0.00586 |
| GO:0003091 | renal water homeostasis | 15 | 2 | 0.12 | 0.00586 |
| GO:0021678 | third ventricle development | 16 | 2 | 0.12 | 0.00666 |
| GO:0001757 | somite specification | 16 | 2 | 0.12 | 0.00666 |
| GO:0043523 | regulation of neuron apoptotic process | 334 | 8 | 2.59 | 0.00684 |
| GO:0031323 | regulation of cellular metabolic process | 4332 | 29 | 33.65 | 0.00846 |
| GO:0048167 | regulation of synaptic plasticity | 300 | 8 | 2.33 | 0.00848 |
| GO:0021592 | fourth ventricle development | 19 | 2 | 0.15 | 0.00935 |
| *RDA Europe* | |  |  |  |  |
| GO:0015808 | L-alanine transport | 16 | 3 | 0.15 | 0.00044 |
| GO:0042723 | thiamine-containing compound metabolic process | 6 | 2 | 0.06 | 0.00132 |
| GO:0033135 | regulation of peptidyl-serine phosphorylation | 318 | 7 | 3.03 | 0.00307 |
| GO:0032868 | response to insulin | 1045 | 11 | 9.95 | 0.00428 |
| GO:0010719 | negative regulation of epithelial to mesenchymal transition | 70 | 4 | 0.67 | 0.00451 |
| GO:0006688 | glycosphingolipid biosynthetic process | 47 | 4 | 0.45 | 0.00554 |
| GO:0007291 | sperm individualization | 38 | 3 | 0.36 | 0.00564 |
| GO:0080129 | proteasome core complex assembly | 40 | 3 | 0.38 | 0.00651 |
| GO:0006868 | glutamine transport | 13 | 2 | 0.12 | 0.00658 |
| GO:0051958 | methotrexate transport | 13 | 2 | 0.12 | 0.00658 |
| GO:0001783 | B cell apoptotic process | 62 | 2 | 0.59 | 0.00951 |

| **Measure** | **Description** | **Year** | **Source** |
| --- | --- | --- | --- |
| BIO1 | Annual mean temperature | 1970-2000 | WorldClim |
| BIO2 | Mean diurnal range | 1970-2000 | WorldClim |
| BIO3 | Isothermality | 1970-2000 | WorldClim |
| BIO4 | Temperature seasonality | 1970-2000 | WorldClim |
| BIO5 | Max temperature of warmest month | 1970-2000 | WorldClim |
| BIO6 | Min temperature of coldest month | 1970-2000 | WorldClim |
| BIO7 | Temperature annual range | 1970-2000 | WorldClim |
| BIO8 | Mean temperature wettest quarter | 1970-2000 | WorldClim |
| BIO9 | Mean temperature of driest quarter | 1970-2000 | WorldClim |
| BIO10 | Mean temperature warmest quarter | 1970-2000 | WorldClim |
| BIO11 | Mean temperature coldest quarter | 1970-2000 | WorldClim |
| BIO12 | Annual precipitation | 1970-2000 | WorldClim |
| BIO13 | Precipitation of wettest month | 1970-2000 | WorldClim |
| BIO14 | Precipitation of driest month | 1970-2000 | WorldClim |
| BIO15 | Precipitation seasonality | 1970-2000 | WorldClim |
| BIO16 | Precipitation of wettest quarter | 1970-2000 | WorldClim |
| BIO17 | Precipitation of driest quarter | 1970-2000 | WorldClim |
| BIO18 | Precipitation of warmest quarter | 1970-2000 | WorldClim |
| BIO19 | Precipitation of coldest quarter | 1970-2000 | WorldClim |
| SST 3mo mean | Mean sea surface temperature for May, June, and July | 2002-2010 | MARSPEC |
| SST max | Long term average of maximum sea surface temperature | 2000-2014 | Bio-oracle |
| Human density | Human population density as a proxy for habitat disturbance | 2000 | NASA NEO |
| Anomalies | Mean of annual temperature anomalies | 1975-2005 | NASA NEO |
| Aquaculture  (N. Am) | Average aquaculture intensity index based on aquaculture sites calculated using AQPress | 2005-2015 | Keyser et al. 2018^2^ |
| Aquaculture (Europe) | Aquaculture intensity index based on aquaculture sites calculated using AQPress for sites in Scotland and Norway | Since 2006 | aquaculture.scotland.gov.uk; kart.fiskeridir.no/akva |

**Supplementary Table 2.** Environmental and anthropogenic measures used to identify potential drivers of population decline using random forest. Description for each measure is provided with the years measured and the data source. Aquaculture intensity index is equivalent to calculations of propagule pressure in previous work^2^.

# Supplementary Table 3. Accuracy of hybrid and pure assignments calculated from Newhybrids simulations for Atlantic salmon populations located in regions where aquaculture is present. Three simulations were performed with three replicates using the wild and farm population to create pure and multigenerational hybrids. Accuracy was determined based on the number of individual correctly assigned to their respective genotype class at a posterior probability greater than 0.75. Pure1 and Pure2 correspond to either the farm or wild population and differ between populations. After simulations were tested for accuracy, hybrid assignments were performed within each population. Individuals that assigned to a hybrid with a posterior probability greater than 0.75 (sum of all hybrid classes) were removed from the LinkNe analyses for estimates of N_e_ over time. The primary class that all hybrids assigned to was backcross-wild (BC-W).

| **Pop** | **Hybrid** | **Pure1** | **Pure2** | **Total loci** | **# Loci in Panel** | **Farm Pop** | **Region** | **Hybrids identified & removed from dataset** | |
| --- | --- | --- | --- | --- | --- | --- | --- | --- | --- |
|  |  |  |  |  |  |  |  | **N** | **Type of hybrid** |
| ABH | 0.930 | 0.933 | 1.000 | 1774 | 250 | BIS | Scotland | 2 | BC-W |
| AWE | 0.957 | 1.000 | 0.990 | 1774 | 250 | BIS | Scotland | 1 | BC-W |
| SNIZ | 0.877 | 1.000 | 0.990 | 1774 | 250 | BIS | Scotland | 1 | BC-W |
| CARN | 0.904 | 0.966 | 1.000 | 1774 | 250 | BIS | Scotland | 0 |  |
| DIO | 0.943 | 1.000 | 0.982 | 1774 | 250 | BIS | Scotland | 0 |  |
| GRU | 0.925 | 0.983 | 0.966 | 1774 | 250 | BIS | Scotland | 2 | BC-W |
| MOID | 0.927 | 1.000 | 0.986 | 1774 | 250 | BIS | Scotland | 2 | BC-W |
| GAR | 0.924 | 0.921 | 0.967 | 1774 | 250 | NLA,CKA | NL | 0 |  |
| GRR | 0.883 | 0.920 | 0.964 | 1774 | 250 | NLA,CKA | NL | 1 | BC-W |
| CNR | 0.951 | 0.983 | 0.958 | 1774 | 250 | NLA,CKA | NL | 1 | BC-W |
| BDN | 0.829 | 0.861 | 0.969 | 1774 | 250 | NLA,CKA | NL | 0 |  |
| LHR | 0.892 | 0.958 | 0.891 | 1774 | 250 | NLA,CKA | NL | 0 |  |
| NHR | 0.998 | 1.000 | 1.000 | 220000 | 250 | CKA | BOF | 0 |  |
| STW | 0.954 | 0.981 | 1.000 | 4357 | 250 | CKA | BOF | 0 |  |
| GAK | 0.956 | 0.965 | 1.000 | 4357 | 250 | CKA | BOF | 0 |  |
| NSH | 1.000 | 1.000 | 1.000 | 220000 | 250 | CKA | BOF | 0 |  |
| BSR | 1.000 | 1.000 | 1.000 | 220000 | 250 | CKA | BOF | 0 |  |
| ***Mean*** | ***0.932*** | ***0.969*** | ***0.980*** |  |  |  |  |  |  |

# Supplementary figures

**
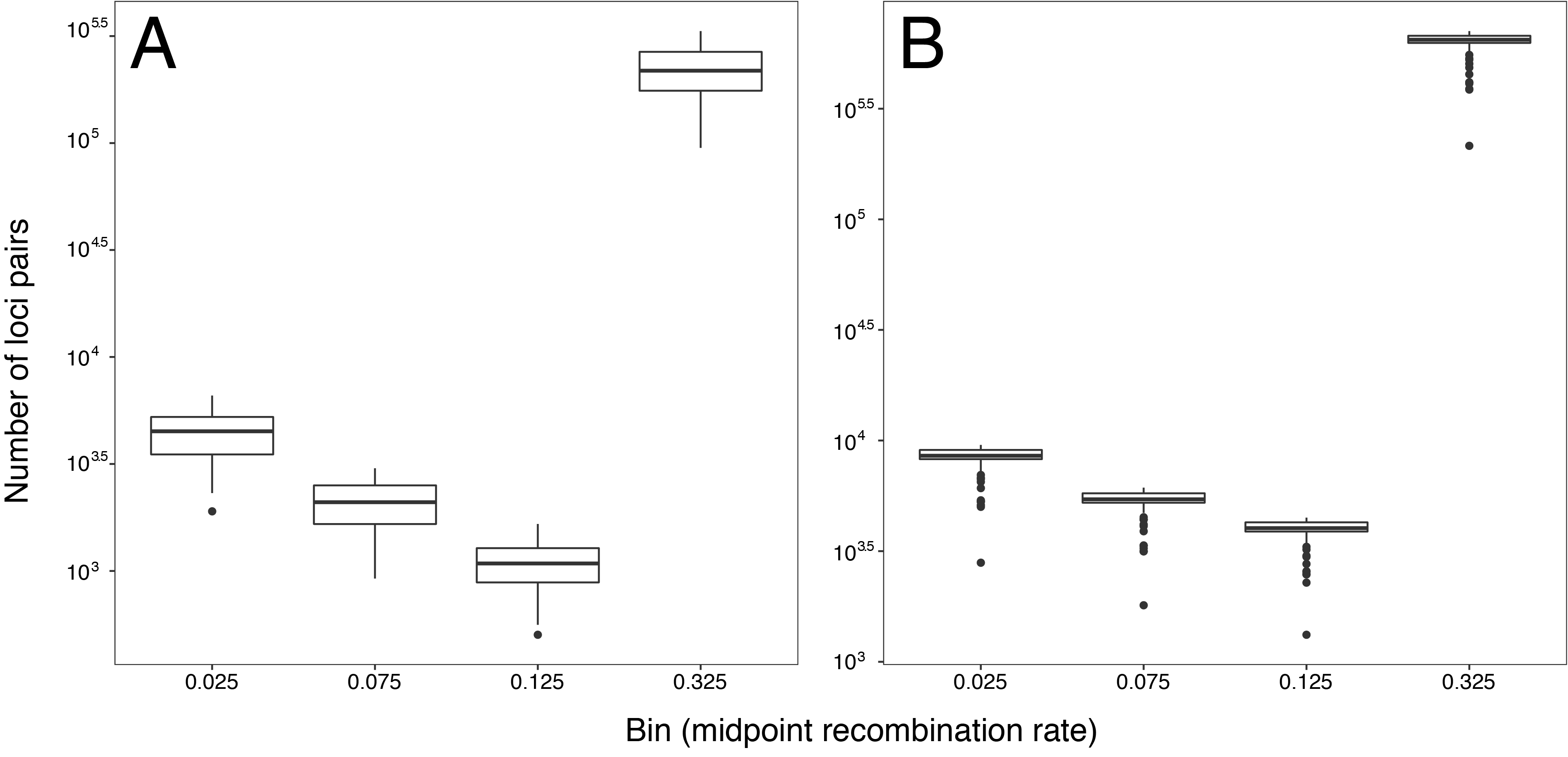
**

**Supplementary Figure 1.** Boxplots for **(A)** North America and **(B)** Europe datasets showing the number of locus pairs used to estimate N_e_ for generations in the past. Bins representing different generations were determined based on recombination rate, where higher recombination rate (*i.e.*, 0.325) reflect the most recent generation. For boxplots, center line indicates median number of loci, box limits represent upper and lower quartiles, whiskers indicate 1.5× interquartile range, and points outside this range are outliers.

**Supplementary Figure 2.** N_e_ estimates with jackknifed confidence interval for three Atlantic salmon populations with historical and contemporary samples. Estimates were determined using the linkage-disequilibrium method in NeEstimator. Asterisk (*) indicates a significant decline in N_e_ between samples based on our classification criteria (see Methods). Classifications based on LinkNe (indicated above panels) are consistent with classifications based on NeEstimator. Populations are Norwegian and include (A) Gaula in Sør-Trøndelag (GauST), (B) Lærdalselva (Laer), and (C) Numedalslågen (Nume).

**
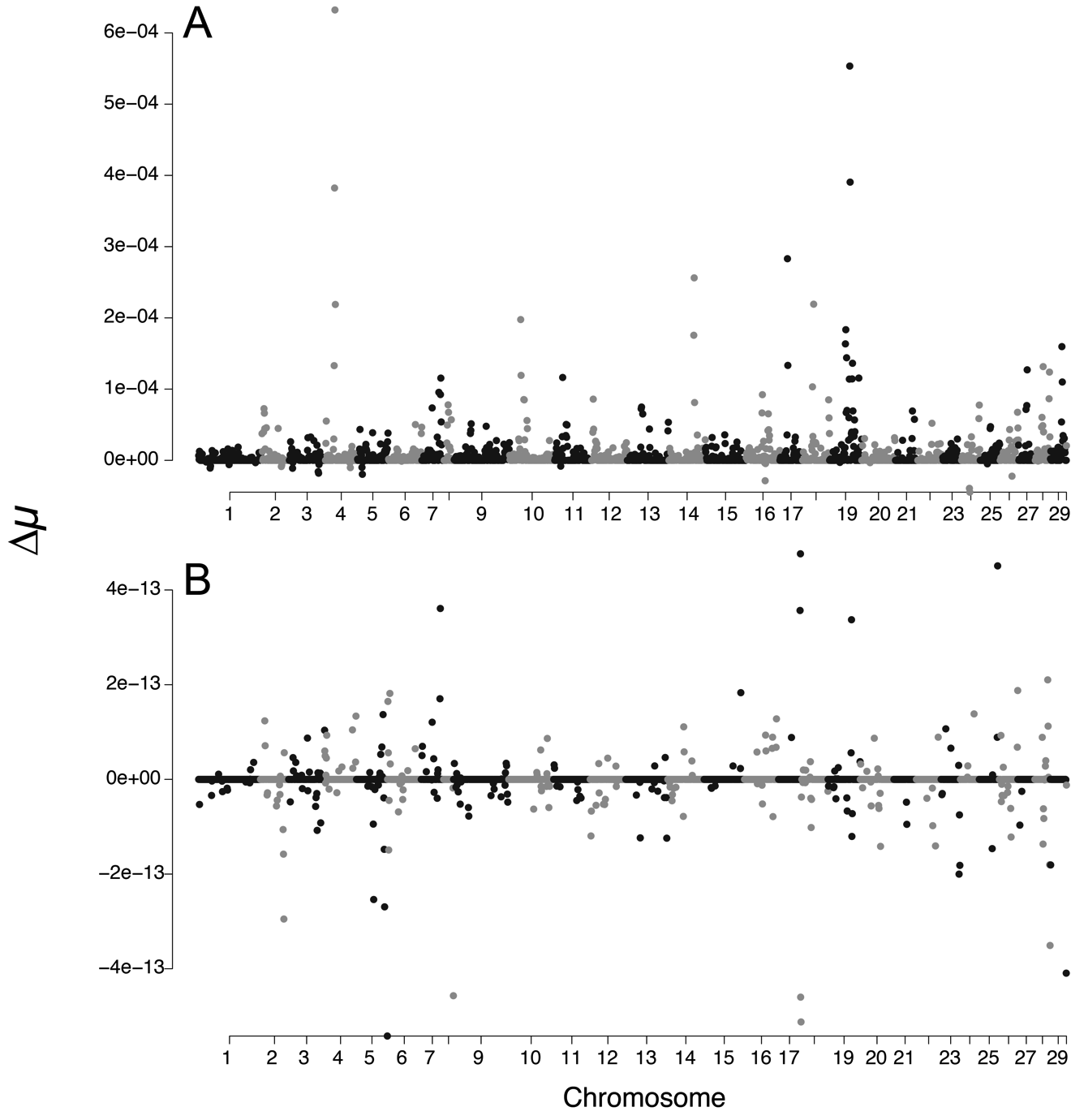
Supplementary Figure 3.** Difference in µ statistic (representing signatures of sweeps) between declining and non-declining populations from overlapping windows across the genome for **(A)** North America and **(B)** Europe. Within each continent, we calculated the change in µ (∆µ) by subtracting µ in declining populations from µ in the non-declining populations, representing the change in selective sweeps (or adaptive diversity) between these groups.

**
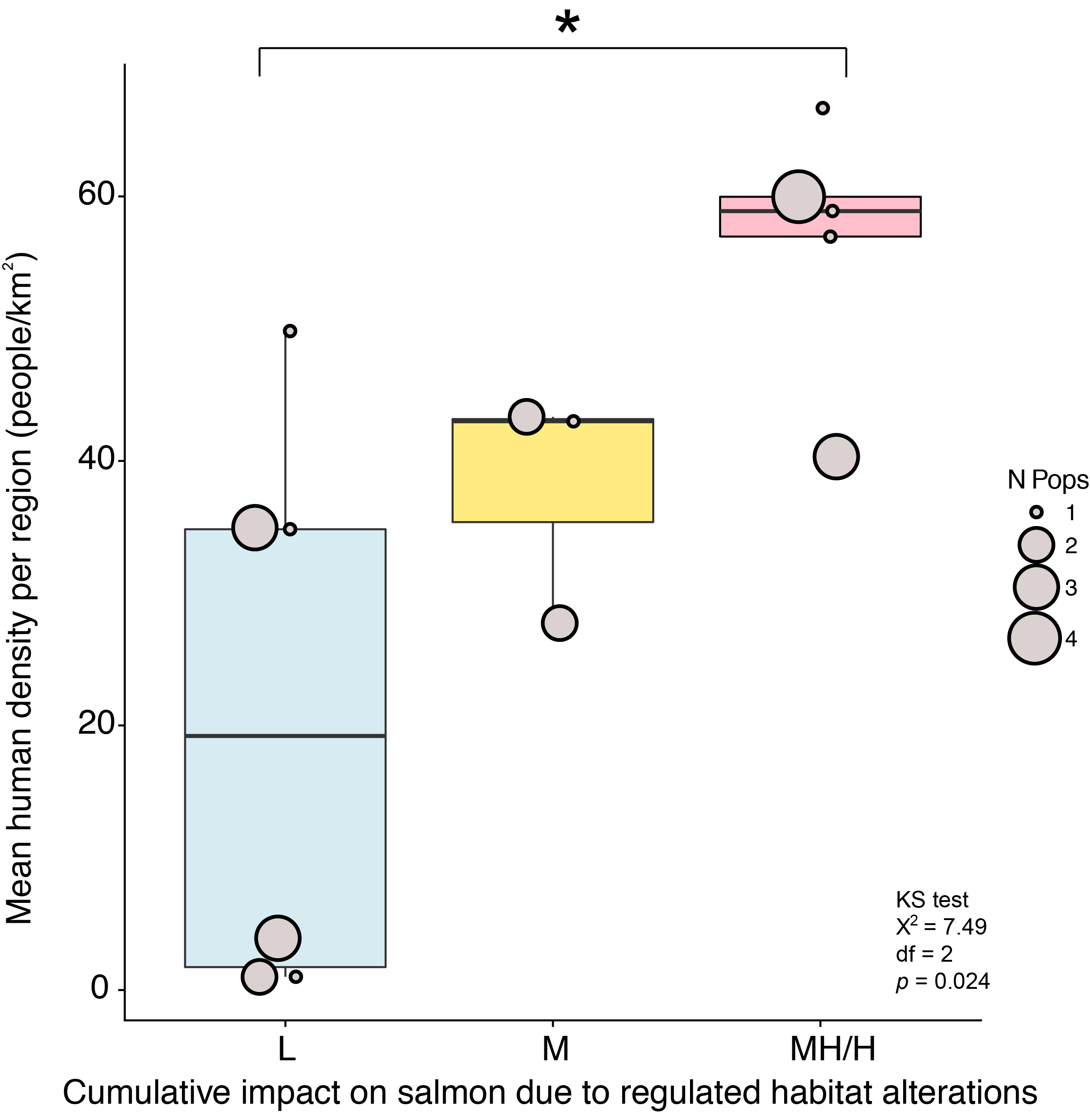
**

**Supplementary Figure 4.** Mean human population density for regions (n=14 regions) of low (L), medium (M), and high (H, and includes regions labelled as MH) impact due to habitat alterations. Impact groups represent different proportion of salmon affected by cumulative effects of regulated habitat alterations, where L, M, and H correspond to <5%, 5-30%, and >30% of salmon affected, respectively. Mean human density was calculated from populations sampled within each region in our study (n=26 populations). Asterisk (*) indicates significant difference between groups. All data points are shown and jittered horizontally. For boxplots, center line indicates median density, box limits represent upper and lower quartiles, whiskers indicate 1.5× interquartile range, and points outside this range are outliers.

**
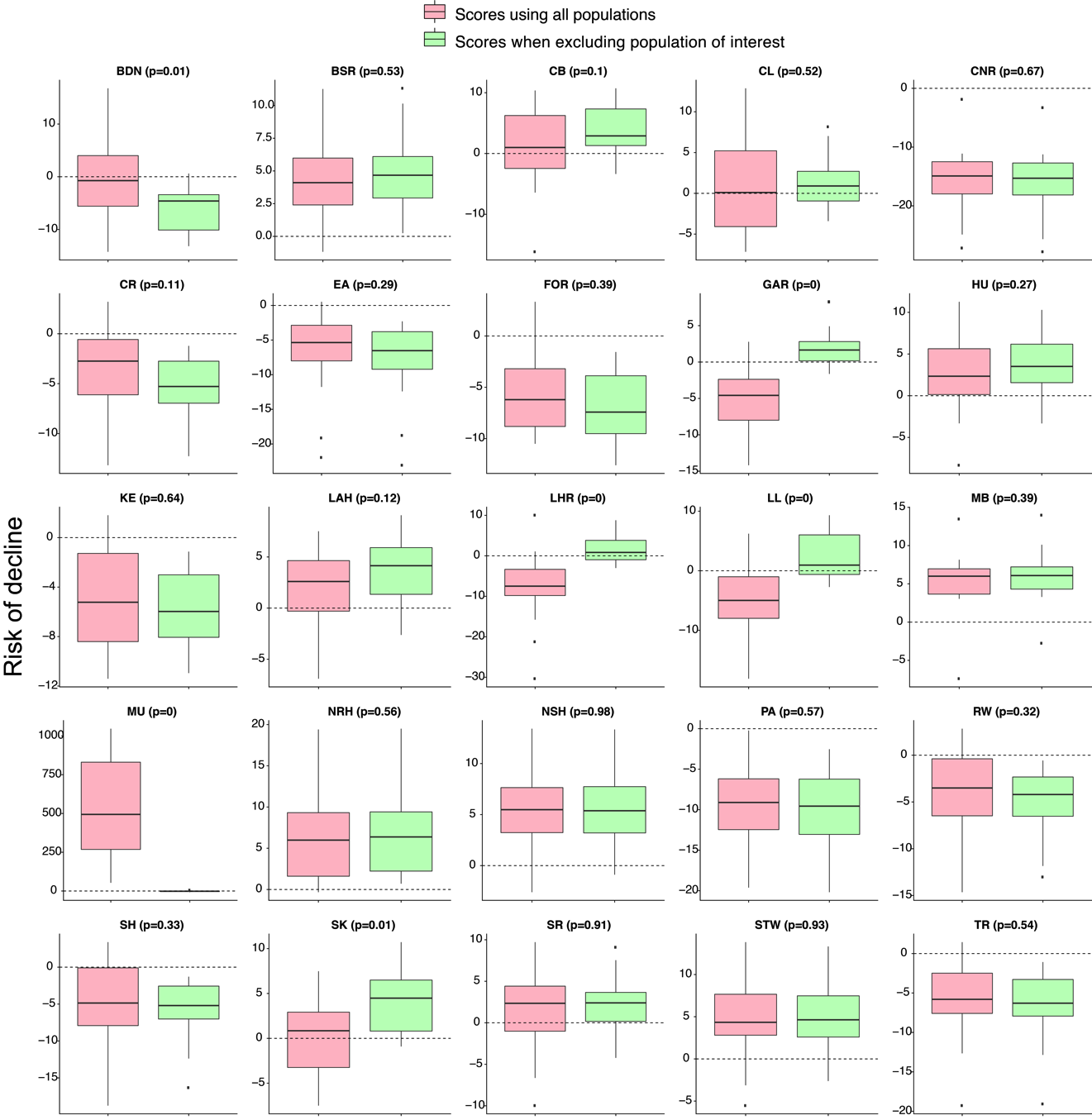
Supplementary Figure 5.** Repeatability of polygenic risk scores for Atlantic salmon populations in North America. Boxplots of polygenic risk scores calculated with all populations (pink) and without the population of interest (green). Population names are listed above plots along with the *p*-value for Mann-Whitney test for comparison between scores when the population of interest was included or excluded in the construction of the risk model. Alpha level was adjusted to determine significant differences (alpha=0.05/25=0.002). For boxplots, center line indicates median risk score, box limits represent upper and lower quartiles, whiskers indicate 1.5× interquartile range, and points outside this range are outliers.


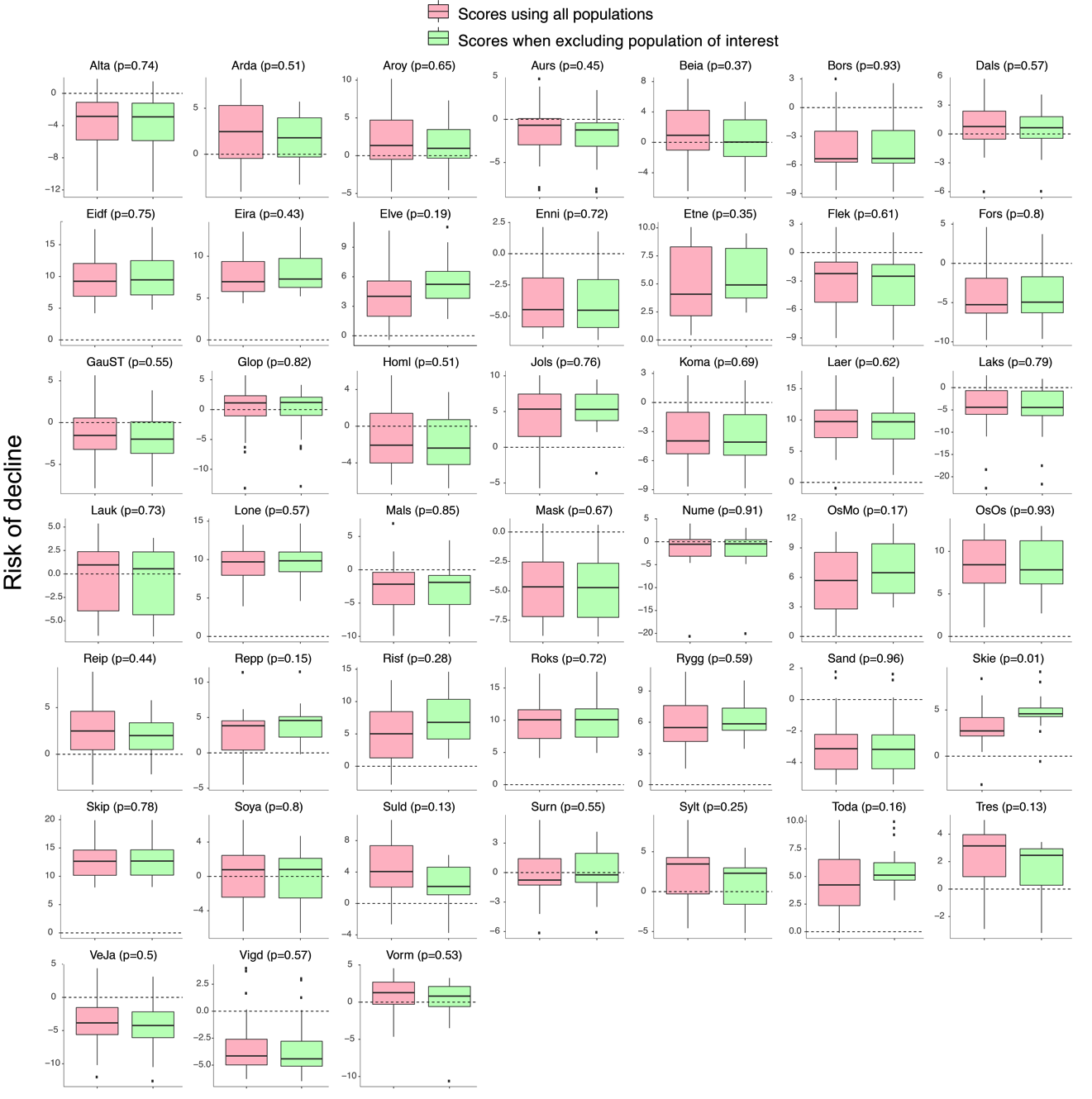
**Supplementary Figure 6.** Repeatability of polygenic risk scores for Atlantic salmon populations in Europe. Boxplots of polygenic risk scores calculated with all populations (pink) and without the population of interest (green). Population names are listed above plots along with the *p*-value for Mann-Whitney test for comparison between scores when the population of interest was included or excluded in the construction of the risk model. Alpha level was adjusted to determine significant differences (alpha=0.05/45=0.001). For boxplots, center line indicates median risk score, box limits represent upper and lower quartiles, whiskers indicate 1.5× interquartile range, and points outside this range are outliers.

**
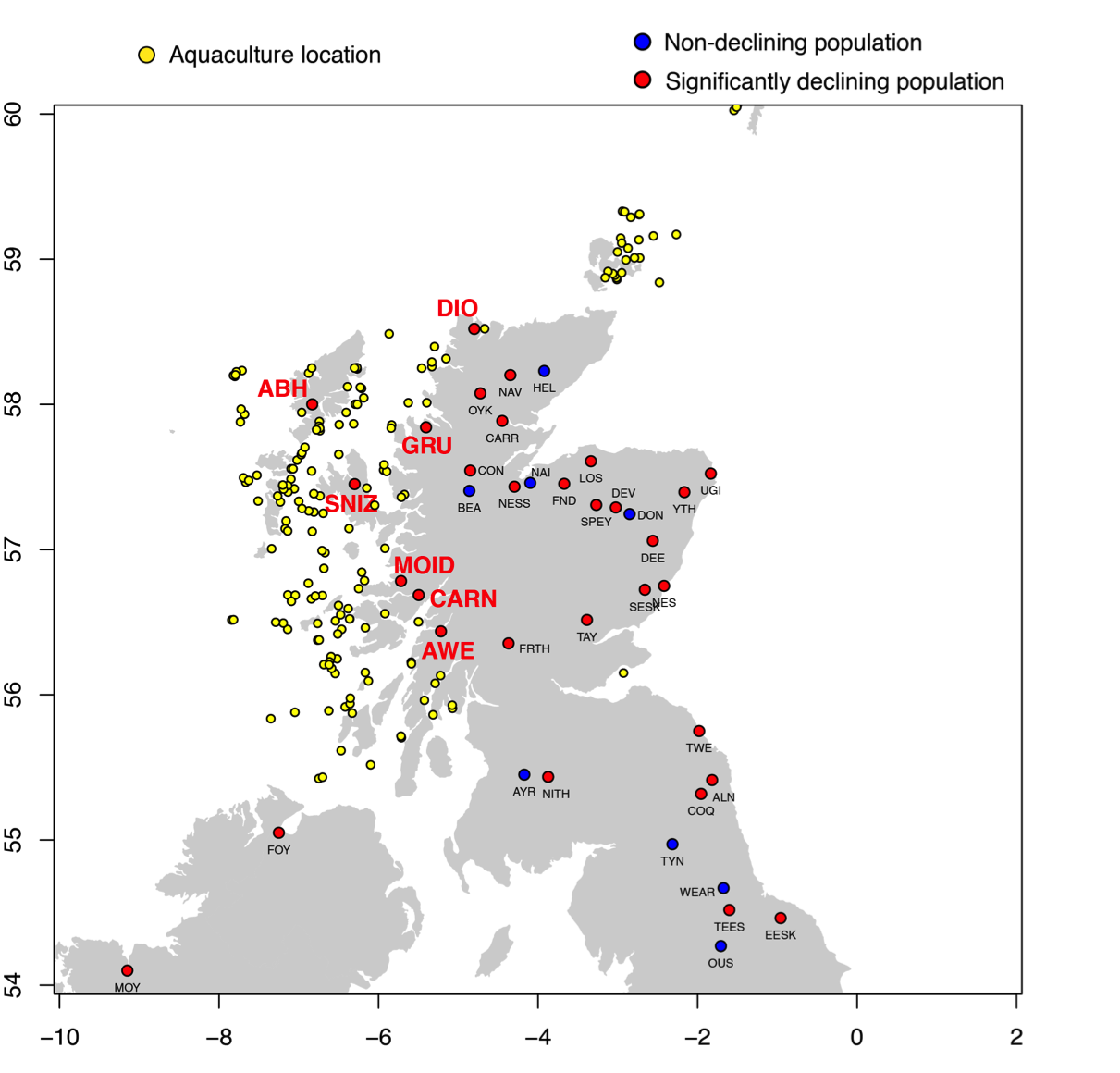
Supplementary Figure 7.** Map of aquaculture sites and sampling sites in Scotland where populations classified as declining or non-declining are indicated in red and blue, respectively. Seven sites located on the western coast of Scotland are highlighted in bold red text and were analysed for introgression from aquaculture provided their close proximity to aquaculture locations.

**
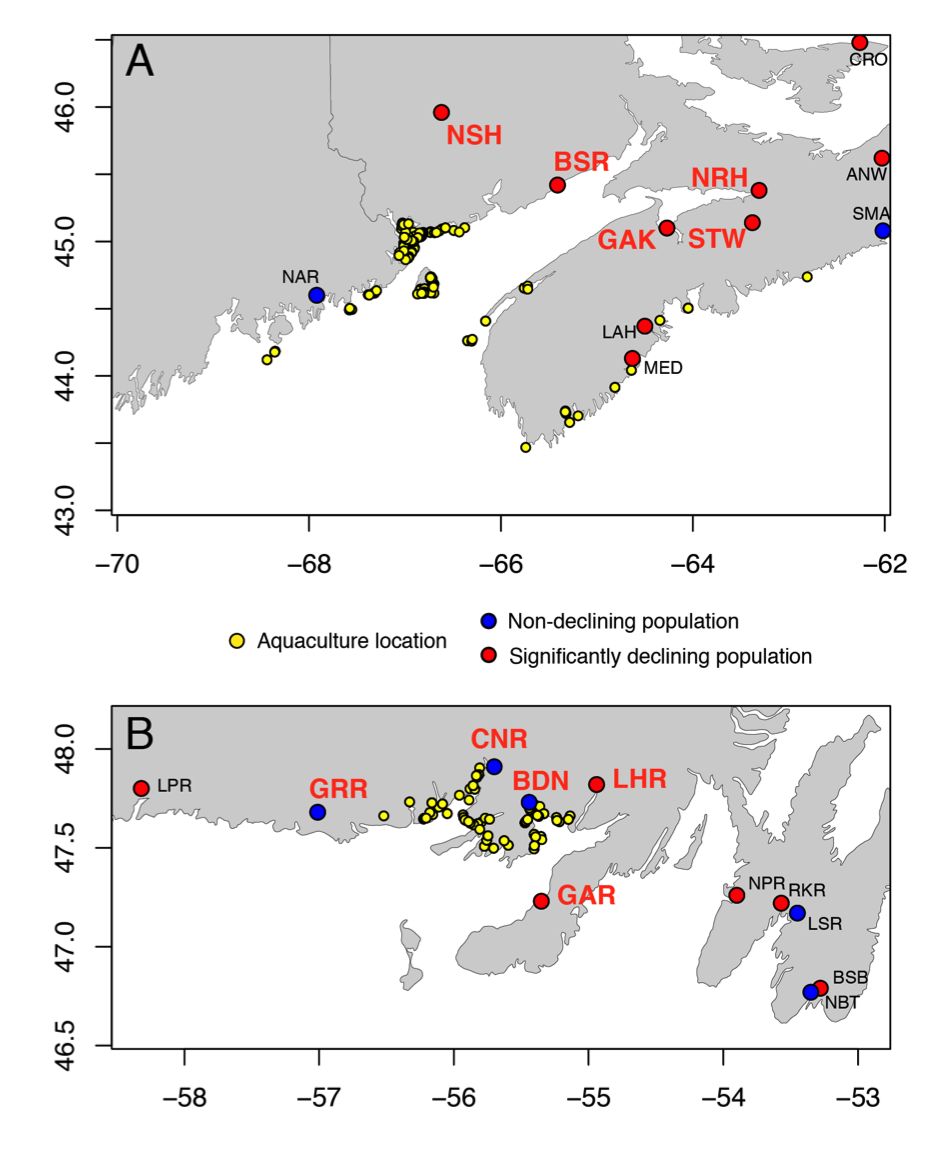
**

**Supplementary Figure 8.** Map of aquaculture sites (yellow) and sampling sites in A) the Bay of Fundy region (New Brunswick and Nova Scotia) and B) southern Newfoundland (NL). Populations classified as declining or non-declining are indicated in red and blue, respectively. Site names highlighted in bold red text were analysed for introgression from aquaculture provided their close proximity to aquaculture locations.

**
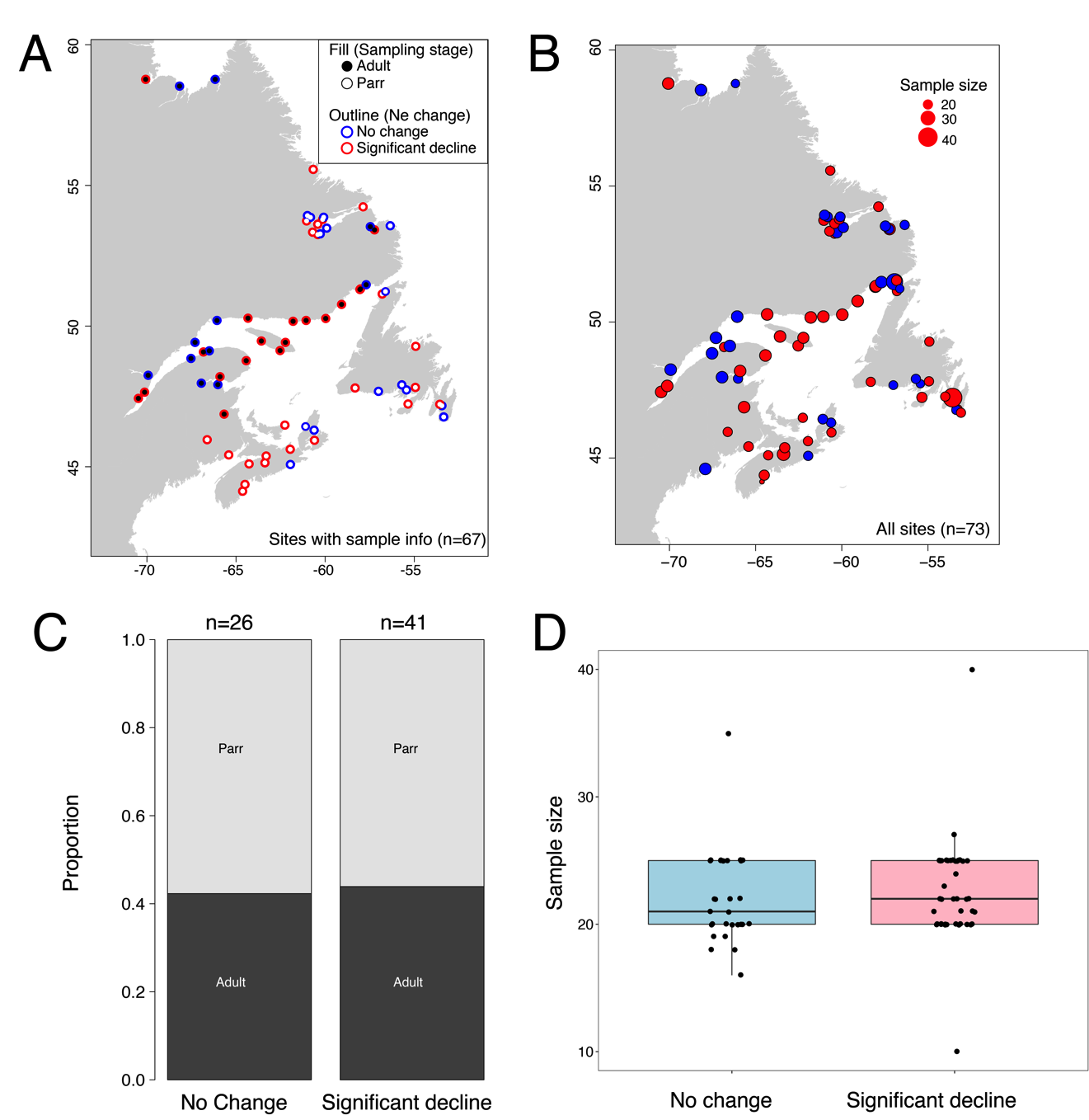
Supplementary Figure 9.** Maps of sites significantly declining (red) and non-declining (blue) populations where (A) shows which life stage was sampled (adult [black] and parr [white]) and (B) shows the sample size for each site. The classification of populations was not dependent on the (C) life stage sampled or (D) the sample size (data points are jittered horizontally). For boxplots, center line indicates median sample size, box limits represent upper and lower quartiles, whiskers indicate 1.5× interquartile range, and points outside this range are outliers.

**
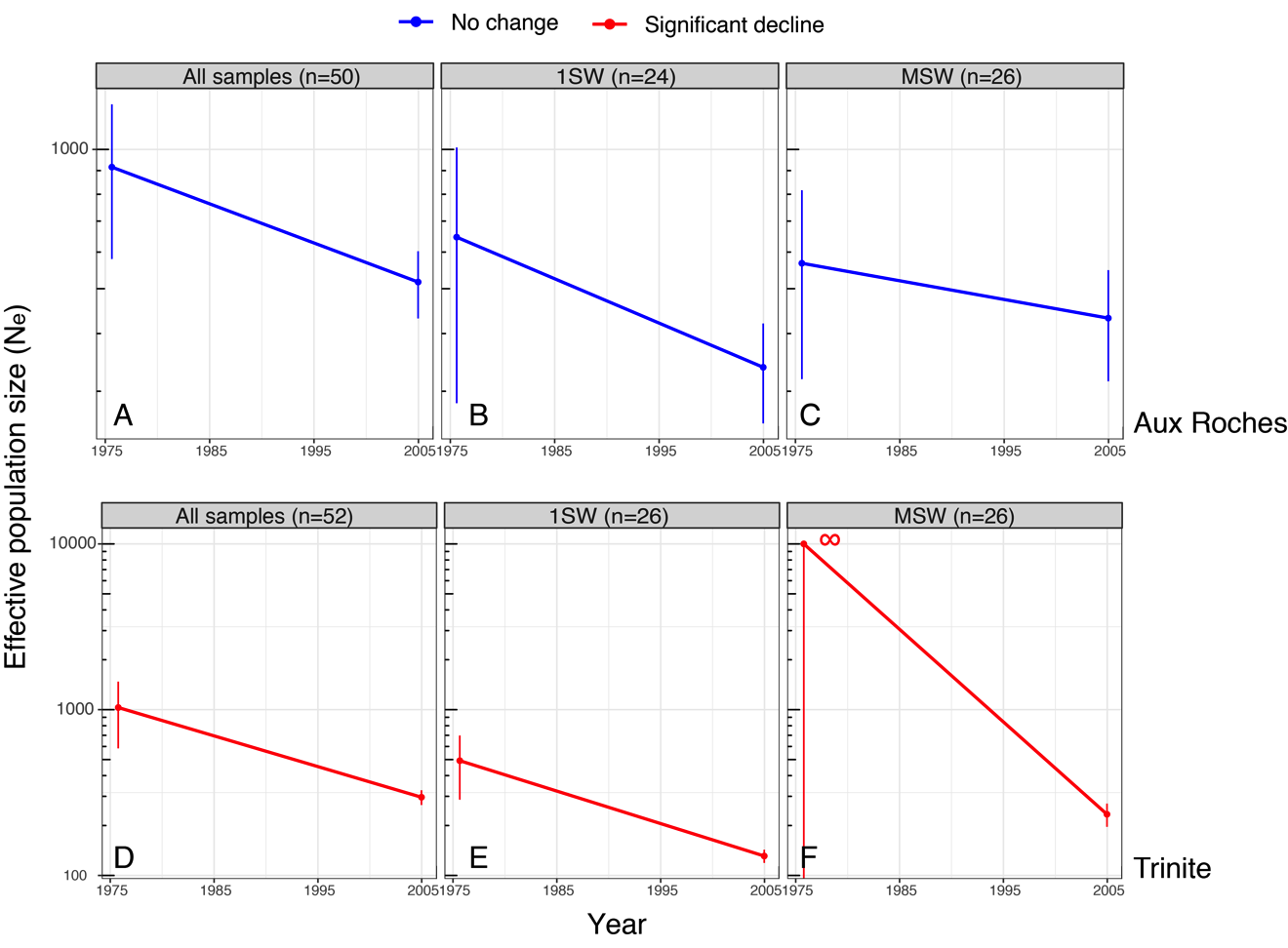
Supplementary Figure 10.** Classifications based on LinkNe results (significantly declining [red] and no change [blue]) for two populations (A-C: Aux Roches; D-F: Trinite). Analyses were performed with all adult samples together (A,D) or separated by age class (B,E: 1-sea winter; C,F: multi-sea winter). Classifications were consistent regardless of sampling strategy. Populations were sampled in 2017 and recently genotyped as part of a different study (not analyzed in the current dataset). Plots show Ne estimate with empirical 95% confidence interval.

**
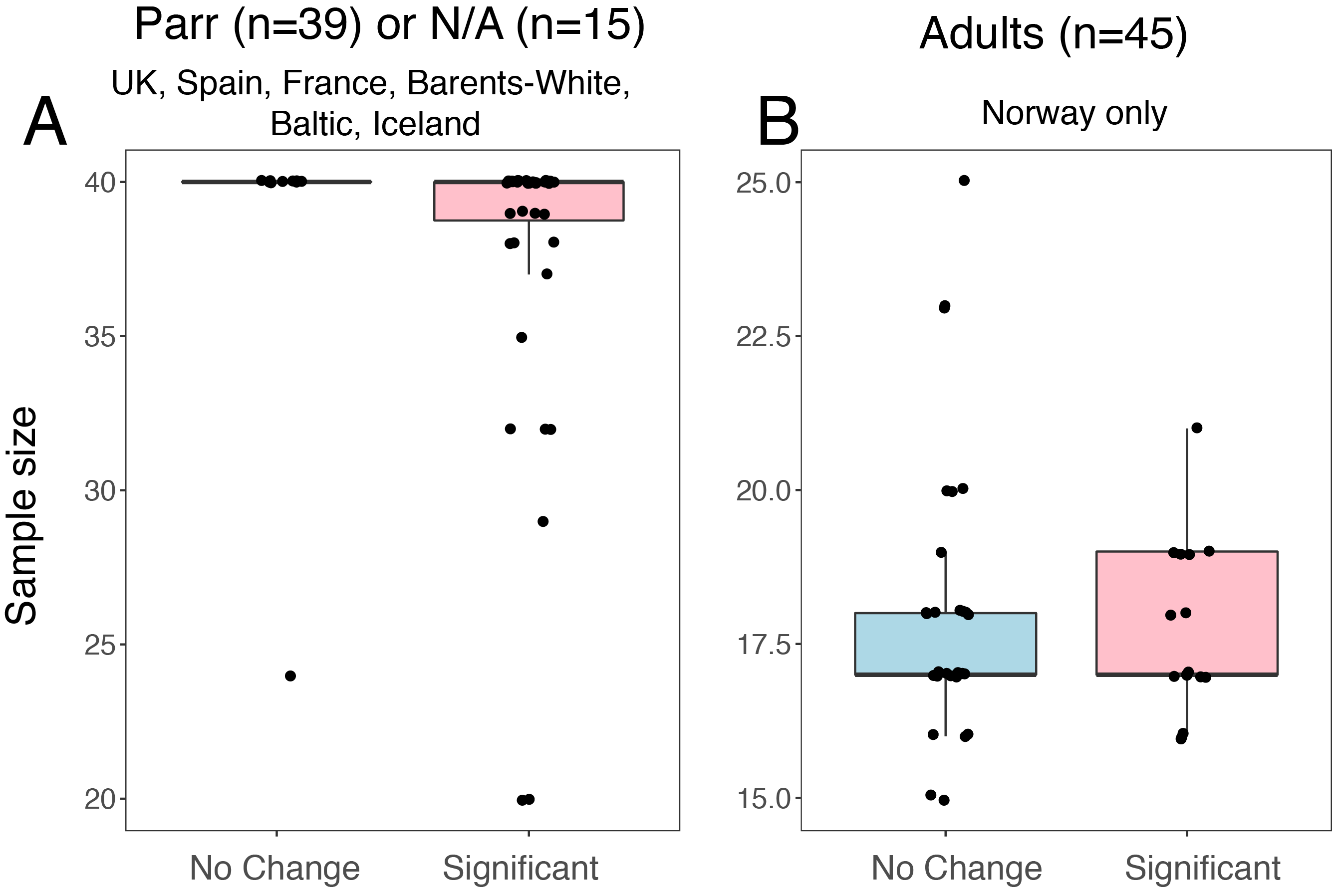
Supplementary Figure 11.** Population classification (significant decline or no decline) and sample size for European populations of Atlantic salmon with samples separated by life stage where (A) shows parr (or unknown) and (B) shows adults. Life stages are shown separately as sampling was dependent on geographic origin. All data points are jittered horizontally. For boxplots, center line indicates median sample size, box limits represent upper and lower quartiles, whiskers indicate 1.5× interquartile range, and points outside this range are outliers.

**Supplementary References**

1. Wringe, B. F. et al. Extensive hybridization following a large escape of domesticated Atlantic salmon in the Northwest Atlantic. *Comm. Biol.* **1**, 108 (2018).

2. Keyser, F. et al. Predicting the impacts of escaped farmed Atlantic salmon on wild salmon populations. *Can. J. Fish. Aquat. Sci.* **75**, 506-512 (2018).

3. Glover, K. A. et al. Atlantic salmon populations invaded by farmed escapees: quantifying genetic introgression with a Bayesian approach and SNPs. *BMC Genet.* **14**, 74 (2013).

4. Green, D. M. et al. The impact of escaped farmed Atlantic salmon (Salmo salar L.) on catch statistics in Scotland. *PLoS ONE* **7**, e43560 (2012).

5. Webb, J. et al. Spawning of escaped farmed Atlantic salmon, *Salmo salar* L., in western and northern Scottish rivers: egg deposition by females. *Aquac. Res.* **24**, 663-670 (1993).

6. Barson, N. J. et al. Sex-dependent dominance at a single locus maintains variation in age at maturity in salmon. *Nature* **528**, 405-408 (2015).

7. Anderson, E. C. Bayesian inference of species hybrids using multilocus dominant genetic markers. *Philos. Trans. R. Soc. Lond. B.* **363**, 2841-2850 (2008).

8. Wringe, B. F., Anderson, E. C., Jeffery, N. W., Stanley, R. R. & Bradbury, I. R. Development and evaluation of SNP panels for the detection of hybridization between wild and escaped Atlantic salmon (*Salmo salar*) in the western Atlantic. *Can. J. Fish. Aquat. Sci.*, 1-10 (2018).

9. Wringe, B. F., Stanley, R. R., Jeffery, N. W., Anderson, E. C. & Bradbury, I. R. *hybriddetective*: a workflow and package to facilitate the detection of hybridization using genomic data in R. *Mol. Ecol. Res.* **17**, e275–e284 (2017).

10. Coulson, M. Report on genetic tool development for distinguishing farmed vs wild fish in Scotland. *Managing Aquaculture Interactions Project* (2013).

11. Jeffery, N. W. et al. Range-wide parallel climate-associated genomic clines in Atlantic salmon. *R. Soc. Open Sci.* **4**, 171394 (2017).

12. Wringe, B. F., Stanley, R. R., Jeffery, N. W., Anderson, E. C. & Bradbury, I. R. parallelnewhybrid: an R package for the parallelization of hybrid detection using newhybrids. *Mol. Ecol. Res.* **17**, 91-95 (2017).

13. O'Reilly, P. T., Carr, J. W., Whoriskey, F. G. & Verspoor, E. Detection of European ancestry in escaped farmed Atlantic salmon, *Salmo salar* l., in the Magaguadavic River and Chamcook Stream, New Brunswick, Canada. *ICES J. Mar. Sci.* **63**, 1256-1262 (2006).

14. Lehnert, S. J. et al. Chromosome polymorphisms track trans-Atlantic divergence and secondary contact in Atlantic salmon. *Mol. Ecol.*, in press (2019).

15. DFO. Review of the science associated with the inner Bay of Fundy Atlantic salmon live gene bank and supplementation programs. in *Canadian Science Advisory Secretariat* 37 (2018).

16. Waples, R. S. & England, P. R. Estimating contemporary effective population size based on linkage disequilibrium in the face of migration. *Genetics* **189**, 633-644 (2011).

17. Hollenbeck, C., Portnoy, D. & Gold, J. A method for detecting recent changes in contemporary effective population size from linkage disequilibrium at linked and unlinked loci. *Heredity* **117**, 207-216 (2016).
