# Supplementary material for: Genomic signatures and correlates of widespread population declines in salmon
Source: Nat Commun. 2019 Jul 5;10:2996. doi: 10.1038/s41467-019-10972-w (PMC6611788; doi:10.1038/s41467-019-10972-w)
Supplement: Supplementary file 4 — Reporting Summary [file 41467_2019_10972_MOESM4_ESM.pdf]

nature research

Corresponding author(s): Lehnert SJ

Last updated by author(s): Jun 5, 2019

Reporting Summary

Nature Research wishes to improve the reproducibility of the work that we publish. This form provides structure for consistency and transparency in reporting. For further information on Nature Research policies, see Authors & Referees and the Editorial Policy Checklist.

Statistics

For all statistical analyses, confirm that the following items are present in the figure legend, table legend, main text, or Methods section.

n/a	Confirmed	
☐	☒	The exact sample size (*n*) for each experimental group/condition, given as a discrete number and unit of measurement
☐	☒	A statement on whether measurements were taken from distinct samples or whether the same sample was measured repeatedly
☐	☒	The statistical test(s) used AND whether they are one- or two-sided<br>*Only common tests should be described solely by name; describe more complex techniques in the Methods section.*
☐	☒	A description of all covariates tested
☐	☒	A description of any assumptions or corrections, such as tests of normality and adjustment for multiple comparisons
☐	☒	A full description of the statistical parameters including central tendency (e.g. means) or other basic estimates (e.g. regression coefficient) AND variation (e.g. standard deviation) or associated estimates of uncertainty (e.g. confidence intervals)
☐	☒	For null hypothesis testing, the test statistic (e.g. *F*, *t*, *r*) with confidence intervals, effect sizes, degrees of freedom and *P* value noted<br>*Give P values as exact values whenever suitable.*
☐	☒	For Bayesian analysis, information on the choice of priors and Markov chain Monte Carlo settings
☐	☒	For hierarchical and complex designs, identification of the appropriate level for tests and full reporting of outcomes
☐	☒	Estimates of effect sizes (e.g. Cohen's *d*, Pearson's *r*), indicating how they were calculated

Our web collection on statistics for biologists contains articles on many of the points above.

Software and code

Policy information about availability of computer code

Data collection	All genotype data were compiled from previous studies. Environmental and anthropogenic data were extracted based on latitude/ longitude of sampling sites from publicly available sources cited within the manuscript. Gene ontology information was compiled from SalmoBase.
Data analysis	R Software (packages cited within), BEDTOOLS, PLINK, LinkNe, Newhybrids, gsi_sim, NeEstimator v2, RAiSD

For manuscripts utilizing custom algorithms or software that are central to the research but not yet described in published literature, software must be made available to editors/reviewers. We strongly encourage code deposition in a community repository (e.g. GitHub). See the Nature Research guidelines for submitting code & software for further information.

Data

Policy information about availability of data

All manuscripts must include a data availability statement. This statement should provide the following information, where applicable:

- Accession codes, unique identifiers, or web links for publicly available datasets
- A list of figures that have associated raw data
- A description of any restrictions on data availability

All genotype data were compiled from previous studies. Genotype data for LinkNe analyses were accessed from https://doi.org/10.5061/dryad.cv20d42 and additional data was added from Norwegian sites (https://doi.org/10.5061/dryad.23h4q). Genotype data from 220K SNP array were compiled from other studies (see https://doi.org/10.5061/dryad.4m5d5m9; https://doi.org/10.5061/dryad.93h33/1). Environmental and anthropogenic data were compiled from multiple sources and compiled data files are available at: https://github.com/SarahLehnert/SalmonDecline. Genomic information for outliers (RDA and sweeps) are also available at the gihub link. No custom scripts were used in these analyses.

Field-specific reporting

Please select the one below that is the best fit for your research. If you are not sure, read the appropriate sections before making your selection.

☐ Life sciences ☐ Behavioural & social sciences ☒ Ecological, evolutionary & environmental sciences

For a reference copy of the document with all sections, see nature.com/documents/nr-reporting-summary-flat.pdf

Ecological, evolutionary & environmental sciences study design

All studies must disclose on these points even when the disclosure is negative.

Study description	Our study uses genotype data for Atlantic salmon that was compiled from previous studies. Our study included a total of 99 European populations (n=2858 individuals) and 73 North American populations (n=1635 individuals). Number of samples for each population are provided in Supplementary Data 1.
Research sample	All genotype data were compiled from previous studies using wild Atlantic salmon (Salmo salar) that were genotyped with a SNP array. Genotype data for LinkNe analyses were accessed from https://doi.org/10.5061/dryad.cv20d42 and https://doi.org/10.5061/dryad.23h4q. Genotype data from 220K SNP array were compiled from other studies (see https://doi.org/10.5061/dryad.4m5d5m9; https://doi.org/10.5061/dryad.93h33/1). Each site location is considered a population.
Sampling strategy	No sampling was performed in our study. We compiled genotype data from previous studies (see Supplementary Data 1) and used all available data. We did considered how sample size and life stage sampled could influence our results. We found no effect of sample size/age on our study outcomes (see Supplementary Note 2: Sample size and age structure; Supplementary Figures 9-11)
Data collection	Genotype data were compiled from previous studies that were genotyped in Atlantic salmon using similar technologies (SNP array).
Timing and spatial scale	Genotype data compiled from previous studies. For the main analyses, we used samples that were collected between 2000 and 2014. These dates were chosen because they represent recent sampling events. These samples were collected from wild Atlantic salmon across Europe and North America. Locations and dates of collection are provided in Supplementary Data 1. Additionally, older samples were also included that were collected from three locations in Norway prior to 1997 (see Supplementary Figure 2) for comparisons between historical and contemporary samples.
Data exclusions	For our main Ne analysis, we excluded collection sites that were sampled prior to the year 2000 because our analyses required recent samples for historical reconstructions of Ne. Data files from Dryad were also checked for duplicate samples. Any redundant samples (i.e., no genotype mismatches) were removed. We also excluded any individuals with evidence of aquaculture ancestry from our Ne analyses because these individuals could potential skew the estimates of Ne. Exclusion of these data are described in the Methods.
Reproducibility	No experiments were performed in our study. Data were compiled from previous studies.
Randomization	This is not relevant to our study, as data were compiled from previous studies.
Blinding	This is not relevant to our study, as data were compiled from previous studies.

Did the study involve field work? ☐ Yes ☒ No

Reporting for specific materials, systems and methods

We require information from authors about some types of materials, experimental systems and methods used in many studies. Here, indicate whether each material, system or method listed is relevant to your study. If you are not sure if a list item applies to your research, read the appropriate section before selecting a response.

Materials & experimental systems

n/a	Involved in the study	
☒	☐	Antibodies
☒	☐	Eukaryotic cell lines
☒	☐	Palaeontology
☐	☒	Animals and other organisms
☒	☐	Human research participants
☒	☐	Clinical data

Methods

n/a	Involved in the study	
☒	☐	ChIP-seq
☒	☐	Flow cytometry
☒	☐	MRI-based neuroimaging

Animals and other organisms

Policy information about studies involving animals; ARRIVE guidelines recommended for reporting animal research

Laboratory animals	The study did not involve laboratory animals.
Wild animals	Genotype data were compiled from previous studies using wild Atlantic salmon (see Supplementary Data 1 for sample details). This study did not sample wild animals.
Field-collected samples	The study did not involve samples collected in the field.
Ethics oversight	No ethical approval was required for this study because no animals were sampled in this study. Data were compiled from previous studies.

Note that full information on the approval of the study protocol must also be provided in the manuscript.
