# Supplementary material for: Genomic signatures and correlates of widespread population declines in salmon
Source: Nat Commun. 2019 Jul 5;10:2996. doi: 10.1038/s41467-019-10972-w (PMC6611788; doi:10.1038/s41467-019-10972-w)
Supplement: Supplementary file 5 — Description of Additional Supplementary Files [file 41467_2019_10972_MOESM5_ESM.docx]

**Title: Supplementary Data 1**
**Description:** European and North American Atlantic salmon populations used to reconstruct estimates of effective population size. Changes in N_e_ are indicated as significant declines (S*) or no significant decline (NS) between 1975 and 2005. Genomic data were compiled from previous studies referenced here (additional studies may be referenced within). The SNP array that was used to genotype individuals is indicated along with the number of individuals (N) used in LinkNe analyses after screening for introgression and redundant samples. Significance of N_e_ declines were determined based on overlap between confidence intervals in 1975 and 2005 for each population (see Methods). Sampling stage when fish were sampled is provided. For some sites, life stage information was not available, but provided the larger sample size (n>30) at many of these sites, juveniles (parr) were likely collected.

**Title: Supplementary Data 2
Description:** Gene ontology enrichment for genes found within outlier sweep windows that show differences between non-declining and declining populations in selective sweeps for North America and Europe. To determine outlier sweep windows, RAiSD was used to calculate the µ statistic (representing signatures of sweeps) across the genome from overlapping windows for declining and non-declining populations separately in each continent. The change in µ (∆µ) was determined by subtracting µ in declining populations from µ in the non-declining populations, representing the difference in adaptive diversity between these groups. Outlier sweep windows were determined by ∆µ values ±3 standard deviations (SD) from the mean. For North America, outlier sweep windows were all characterized by positive ∆µ, whereas in Europe both negative and positive ∆µ outlier sweep windows were found and results are provided separately in the table. The numbers of genes with GO annotations are provided for both the reference dataset (N_anno_) and outlier dataset (N_sig_) along with the expected number (N_exp_) based on a random distribution and significance (p-val) using Fisher’s exact test. An alpha level of 0.01 was used to determine significance.
